# Iron Deficiency Modulates Metabolic Landscape of *Bacteroidetes* Promoting Its Resilience during Inflammation

**DOI:** 10.1128/spectrum.04733-22

**Published:** 2023-06-14

**Authors:** Janina P. Lewis, Qin Gui

**Affiliations:** a Philips Institute for Oral Health Research, Virginia Commonwealth University, Richmond, Virginia, USA; b Department of Microbiology and Immunology, Virginia Commonwealth University, Richmond, Virginia, USA; c Department of Biochemistry, Virginia Commonwealth University, Richmond, Virginia, USA; University of Minnesota Twin Cities

**Keywords:** *Bacteroides thetaiotaomicron*, *Bacteroidetes*, *Porphyromonas gingivalis*, *Prevotella intermedia*, host immune response, iron metabolism, iron regulation, iron uptake

## Abstract

Bacteria have to persist under low iron conditions in order to adapt to the nutritional immunity of a host. Since the knowledge of iron stimulon of *Bacteroidetes* is sparse, we examined oral (Porphyromonas gingivalis and Prevotella intermedia) and gut (Bacteroides thataiotaomicron) representatives for their ability to adapt to iron deplete and iron replete conditions. Our transcriptomics and comparative genomics analysis show that many iron-regulated mechanisms are conserved within the phylum. They include genes upregulated in low iron, as follows: *fldA* (flavodoxin), *hmu* (hemin uptake operon), and loci encoding ABC transporters. Downregulated genes were *frd* (ferredoxin), *rbr* (rubrerythrin), *sdh* (succinate dehydrogenase/fumarate reductase), *vor* (oxoglutarate oxidoreductase/dehydrogenase), and *pfor* (pyruvate:ferredoxin/flavodoxin oxidoreductase). Some genus-specific mechanisms, such as the *sus* of B. thetaiotaomicron coding for carbohydrate metabolism and the *xusABC* coding for xenosiderophore utilization were also identified. While all bacteria tested in our study had the *nrfAH* operon coding for nitrite reduction and were able to reduce nitrite levels present in culture media, the expression of the operon was iron dependent only in B. thetaiotaomicron. It is noteworthy that we identified a significant overlap between regulated genes found in our study and the B. thetaiotaomicron colitis study (W. Zhu, M. G. Winter, L. Spiga, E. R. Hughes et al., Cell Host Microbe 27:376–388, 2020, http://dx.doi.org/10.1016/j.chom.2020.01.010). Many of those commonly regulated genes were also iron regulated in the oral bacterial genera. Overall, this work points to iron being the master regulator enabling bacterial persistence in the host and paves the way for a more generalized investigation of the molecular mechanisms of iron homeostasis in *Bacteroidetes*.

**IMPORTANCE** *Bacteroidetes* are an important group of anaerobic bacteria abundant both in the oral and gut microbiomes. Although iron is a required nutrient for most living organisms, the molecular mechanisms of adaptation to the changing levels of iron are not well known in this group of bacteria. We defined the iron stimulon of *Bacteroidetes* by examination of the transcriptomic response of Porphyromonas gingivalis and Prevotella intermedia (both belong to the oral microbiome) and Bacteroidetes thetaiotaomicron (belongs to the gut microbiome). Our results indicate that many of the iron-regulated operons are shared among the three genera. Furthermore, using bioinformatics analysis, we identified a significant overlap between our *in vitro* studies and transcriptomic data derived from a colitis study, thus underscoring the biological significance of our work. Defining the iron-dependent stimulon of *Bacteroidetes* can help to identify the molecular mechanisms of iron-dependent regulation as well as better understand the persistence of the anaerobes in the human host.

## INTRODUCTION

Iron is emerging as a critical component of the microbiome-liver fat axis (1, 2). Furthermore, iron plays a role in the development of periodontitis through its contribution to the selection of anaerobic pathogenic bacteria in the periodontal pocket (3, 4). The above conditions are associated with increased levels of bacteria belonging to the *Bacteroidetes* phylum. The *Bacteroidetes* phylum are one of the four most abundant phyla in both the oral cavity (5–7) and the gut (8–11). The main characteristic is that those bacteria can grow under microaerophilic/anaerobic conditions and they rely on iron-based metabolism for energy generation (12). Indeed, the central enzyme, pyruvate ferredoxin oxidoreductase (PFOR) (13), which is required for the decarboxylation of pyruvate to acetyl-coenzyme A (CoA), contains four iron-sulfur clusters peripherally located on the surface of the enzyme that are easily oxidized and deactivated under atmospheric oxygen levels (13). That group of bacteria also contains many other enzymes mediating metabolic functions that are critically dependent on iron. Thus, it is intuitive that iron levels would play a central role in the regulation of bacterial metabolism.

Thus far, we have focused our efforts of determination of iron homeostasis on the oral bacterium Porphyromonas gingivalis. This bacterium is an oral pathogen shown to play a role in the development and progression of periodontal disease. We have identified the major hemin uptake locus *hmu* (14) as well as reported on the iron stimulon of the bacterium (15). We also expanded our studies onto the related oral bacterium Prevotella intermedia (16) where we found similar genes/proteins as in P. gingivalis that were deregulated depending on iron levels. However, no comprehensive approaches were undertaken to investigate changes in gene regulation in response to iron levels in P. intermedia. Furthermore, the iron-dependent transcriptome of the intestinal *Bacteroidetes* is yet to be determined. So far, the outer membrane proteins of Bacteroides fragilis and Bacteroides vulgatus were investigated for differential expression; however, no comprehensive approach to determine iron-dependent differential gene expression has been reported (17). Finally, the molecular mechanisms as well as how the iron-dependent regulation in oral *Bacteroidetes* compares to iron-dependent regulation in related bacteria residing in the gut are yet to be determined.

Here we used transcriptome sequencing (RNAseq) followed by comparative genomics to determine the iron stimulon in the following three *Bacteroidetes* species: Bacteroides thetaiotaomicron, P. gingivalis, and P. intermedia. We demonstrate that there is a significant overlap in adaptation to the iron-rich and iron-deficient conditions as evidenced by a common set of genes regulated in all the bacteria studied. Unique mechanisms were also detected and were confined mainly to the B. thetaiotaomicron bacterium.

## RESULTS

### Iron-dependent stimulon of P. gingivalis.

**FIG 1:**
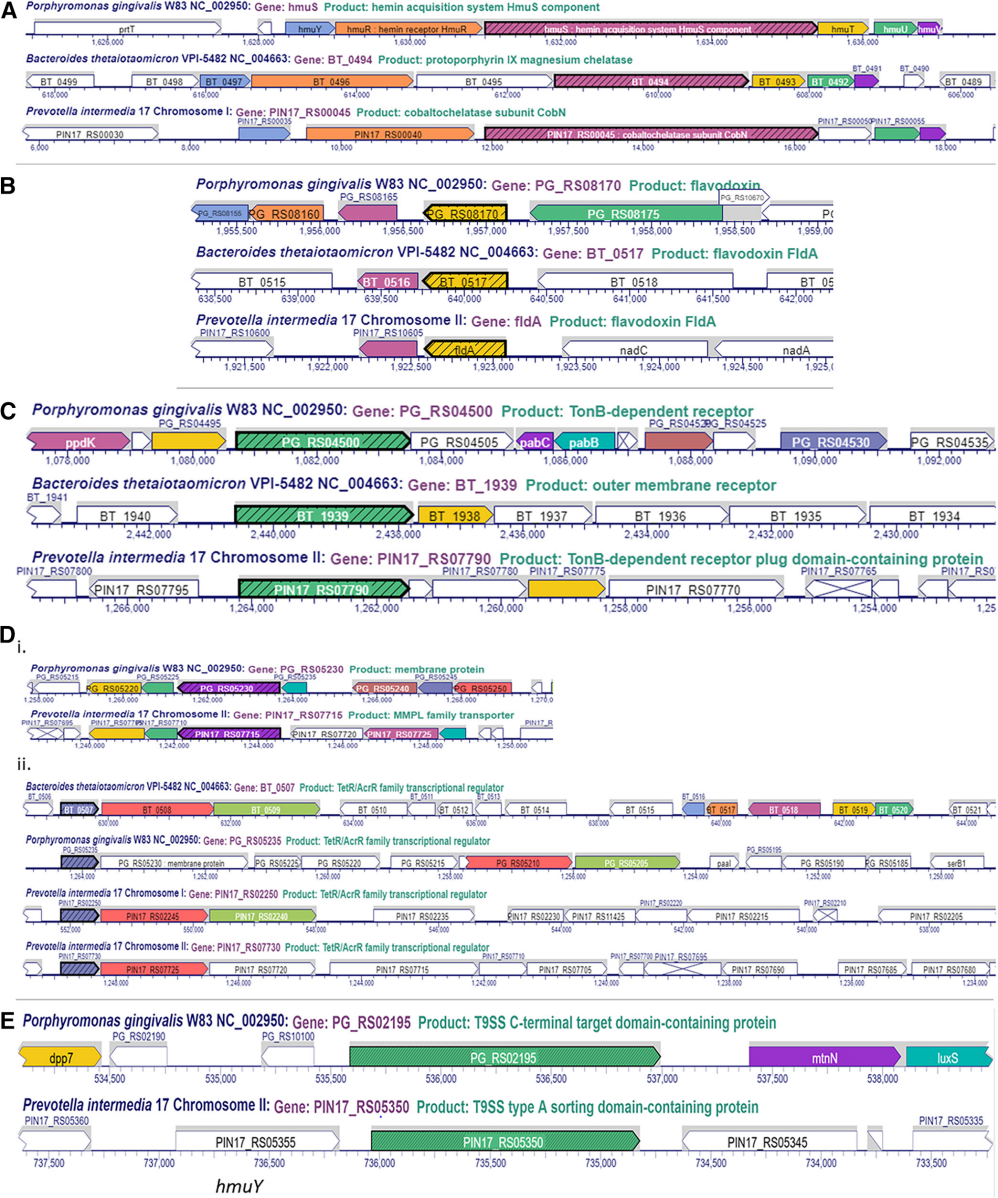
Comparison of loci upregulated in *Bacteroides*. (A) Hemin uptake *hmu* locus in *Bacteroidetes*. Comparison of genomic locus in P. gingivalis W83 (*hmuY* to *hmuT*, PG_RS0), B. thetaiotaomicron VPI-5482 (BT_0491 to PG_RS0598), and P. intermedia 17 (PIN17_RS00035-55). (B) Flavodoxin *fld* locus in *Bacteroidetes* (designated in yellow). Comparison of genomic locus in P. gingivalis W83 (PG_RS08170, PG1858), B. thetaiotaomicron VPI-5482 (BT_0517), and P. intermedia 17 (*fldA*). (C) Outer membrane transport system locus in *Bacteroidetes*. Comparison of genomic locus P. gingivalis W83 (PG_RS04495 to PG_RS04505; PG1019 to 21), B. thetaiotaomicron VPI-5482 (BT_1938 to 9), and P. intermedia 17 (PIN17_RS07775 to 90). (D) TetR/AcrR regulator and transport system locus in *Bacteroidetes*. Comparison of genomic locus P. gingivalis W83 (PG_RS05220 to 235; PG1178 to 81), B. thetaiotaomicron VPI-5482 (BT_0507-9), and P. intermedia 17 (PIN17_RS07705 to 25, PIN17_RS02240 50, PIN17_RS07725 to 30). (E) Cell surface extracellular protein in *Bacteroidetes*. Comparison of genomic locus P. gingivalis W83 (PG_RS02195, PG0495), B. thetaiotaomicron VPI-5482 (no ortholog found), and P. intermedia 17 locus (PIN_RS05350).

Genes that were the most upregulated in P. gingivalis W83 grown under iron-depleted conditions compared with iron-replete conditions are listed in Table 1. Genomic loci of selected genes are also shown in Fig. 1. A total of 96 genes were upregulated 1.4-fold or more. Among those genes, 11 operons were identified. The most highly upregulated gene was PG0827 coding for a MATE family efflux transporter (920-fold upregulation). This gene has been reported to also have *cis*-encoded antisense transcript indicating that it may be regulated by small RNA (18). The PG0495 coding for a T9SS type A sorting domain-containing protein was upregulated 87-fold. Also, several transporter-encoding operons were upregulated and included, as follows: the PG1551 to PG15516 operon encoding the Hmu hemin uptake system (upregulated 11.4- to 30.1-fold), PG1175 to PG1181 coding for a two-component lipoprotein/MMPL transporter followed by ABC transporter ATP-binding proteins as well as the TetR/AcrR family transcriptional regulator (upregulated 13.4- to 136.2-fold), and the PG1019 to PG1022 TonB-dependent transporter system (upregulated by 20.8- to 214.5-fold, depending on a gene). It is noteworthy that genes coding for proteins mediating metabolic processes were also regulated. The most highly upregulated one in this class was the PG1857 to PG1858 operon coding for proteins with unknown function (PG1857, 48–fold) and flavodoxin (PG1858, 133-fold). Also, the locus carrying PG1461 to PG1469 and encoding hypothetical proteins and putative isoprenylcysteine, carboxymethyltransferase family proteins as well as class I SAM-dependent methyltransferase were upregulated by 2- to 27-fold, depending on the gene. Genes encoding proteins that associate with iron, such as the PG1236 to PG1238 operon coding for a hemerythrin domain-containing protein (RluA family pseudouridine synthase) and a response regulator/transcription factor, were upregulated by 2- to 7-fold, depending on the gene. Slightly upregulated were genes encoding iron (FeoB2, PG1294) and manganease (FeoB1, PG1043) transport as well as the *ahpC* gene coding for peroxiredoxin (2.4-fold). Finally, genes encoding the T9SS type A sorting domain-containing protein, PG0654 and PG1374, were upregulated 1.5- and 7.3-fold, respectively.

**TABLE 1:** Genes upregulated in P. gingivalis W83 at least 1.4-fold under iron-depleted conditions^*a*^

Fold change^*b*^	*P* value^*c*^	Gene locus_tag	Old_locus_tag	Product
4.4	0.336	PG_RS10350		Hypothetical protein
2.8	0.241	PG_RS10910		DUF1661 domain-containing protein
2.5	0.174	PG_RS11125		DUF1661 domain-containing protein
1.9	0.316	PG_RS11315		Hypothetical protein
1.8	0.040	PG_RS05700		FeoB-associated Cys-rich membrane protein
1.6	0.038	PG_RS00305		Hypothetical protein
1.5	0.441	PG_RS10915		DUF1661 domain-containing protein
1.5	0.100	PG_RS00455		Hypothetical protein
1.4	0.538	PG_RS09100		IS*5* family transposase
1.6	0.030	PG_RS00020	PG0004	NAD-dependent deacylase
1.6	0.035	PG_RS01345	PG0300	Tetratricopeptide repeat protein
1.5	0.026	PG_RS01395PG_RS01400	PG0311, PG0312	Glycosyltransferase family 2 protein, DUF4199 domain-containing protein
1.4	0.062	PG_RS01535	PG0345	Hypothetical protein
2.8	5.41712E-08	PG_RS01560	PG0350	Leucine-rich repeat domain-containing protein
1.4–1.5	0.031–0.081	PG_RS01620–30	PG0364–66	Class I SAM-dependent rRNA methyltransferase, 3′–5′ exonuclease domain-containing protein 2, DUF5063 domain-containing protein
1.4	0.168	PG_RS01865	PG0419	DUF2807 domain-containing protein
1.7	0.026	PG_RS01945	PG0437	Polysaccharide biosynthesis/export family protein
1.5	0.056	PG_RS02085	PG0469	Hypothetical protein
2.3	0.007	PG_RS02165	PG0487	IS*4*-like element IS1598 family transposase
87.1	0	PG_RS02195	PG0495	T9SS type A sorting domain-containing protein
2.4	0.0005	*ahpC* PG_RS02725	PG0618	Peroxiredoxin
1.5	0.014	PG_RS02890	PG0654	T9SS type A sorting domain-containing protein
2.3	1.74918E-05	PG_RS03020	PG0686	PAS domain-containing protein
2.0	0.0002	PG_RS03105	PG0707	TonB-dependent receptor
1.5	0.099	PG_RS03365	PG0768	DUF2891 domain-containing protein
1.4	0.07–0.078	PG_RS03400–05, *tsaB*	PG0777–78	Electron transfer flavoprotein subunit beta/FixA family protein, tR NA (adenosine(37)-N6)-threonylcarbamoyltransferase complex dimerization subunit type 1 TsaB
11.0	0.083	PG_RS03595	PG0821	Hypothetical protein
920.6	3.36987E-06	PG_RS03625, PG_RS03630	PG0827	MATE family efflux transporter, hypothetical protein
1.8	0.090	PG_RS04015	PG0914	Hypothetical protein
1.4	0.042	PG_RS04045	PG0920	Polyprenol monophosphomannose synthase
1.7	0.006	PG_RS04080	PG0928	PglZ domain-containing protein
1.9	0.045	PG_RS10280	PG0943, PG_0943	IS*3*-like element ISPg5 family transposase
1.4–1.7	0.005–0.101	PG_RS04350–60	PG0985–87	Sigma-70 family RNA polymerase sigma factor, hypothetical protein, DUF4252 domain-containing protein
214.5–20.8	0	PG_RS04495–505	PG1019–22	DUF4876 domain-containing protein, TonB-dependent receptor, hypothetical proteins
3.3–1.6	9.25226E-09–0.008	PG_RS04595–605, *feoB1*	PG1042–44	Glycosyltransferase, ferrous iron transport protein B, DtxR family transcriptional regulator
1.5	0.068	PG_RS04645	PG1055	Thiol protease
1.4	0.057	PG_RS04960	PG1119	NAD(P)H-dependent oxidoreductase
1.5	0.044	PG_RS05005	PG1129	Adenosylcobalamin-dependent ribonucleoside-diphosphate reductase
13.4–136.2	0	PG_RS05205–235	PG1175–81	ABC transporter ATP-binding proteins, IS*5* family transposase, hypothetical protein, outer membrane lipoprotein-sorting protein, MMPL family transporter, TetR/AcrR family transcriptional regulator
6.9–7.3	0	PG_RS05435–40	PG1236–38	Hemerythrin domain-containing protein, response regulator transcription factor
1.9	0.002	PG_RS05445	PG1238	RluA family pseudouridine synthase
1.5	0.025	PG_RS05650	PG1281	DUF2027 domain-containing protein
2.2	1.77345E-05	*feoB2,* PG_RS05695	PG1294	Ferrous iron transport protein B
1.8	0.042	PG_RS05705	PG1296	Hypothetical protein
1.4	0.008	PG_RS05870, PG_RS05875	PG1333, PG1334	Lipid A deacylase, SPFH/Band 7/PHB domain protein
7.3	0	PG_RS06055	PG1374	T9SS type A sorting domain-containing protein
1.5	0.077	PG_RS06085	PG1383	LysE family transporter
1.4	0.042	PG_RS06195, PG_RS06200	PG1408, PG1409	Cation transporter, hypothetical protein
2.0–27.3	0–0.207	PG_RS06415–55	PG1461–69	Hypothetical protein
1.6	0.275	PG_RS06510	PG1480	DUF4141 domain-containing protein
11.4–30.1	0–1.82188E-13	*hmuYRSTUV* PG_RS06840–65	PG1551–56	HmuY family protein, TonB-dependent receptor, cobaltochelatase subunit CobN, hypothetical protein, MotA/TolQ/ExbB proton channel family protein, DUF2149 domain-containing protein
1.4	0.062	PG_RS07200	PG1635	Hypothetical protein
1.4	0.132	PG_RS08040	PG1823	PorT family protein
1.5	0.034	PG_RS08065	PG1829	AMP-binding protein
1.8–133.6	0–0.005	PG_RS08150–70, *fldA*	PG1854–58	5-Formyltetrahydrofolate cyclo-ligase, S41 family peptidase, dCMP deaminase family protein, DUF2023 family protein, flavodoxin
3.7	3.00015E-07	PG_RS08200	PG1868	Isoprenylcysteine carboxyl methyltransferase family protein
15.8	0	PG_RS08205	PG1870	Class I SAM-dependent methyltransferase
1.5–1.6	0.033–0.065	PG_RS08235–40	PG1878–79	Cysteine-tRNA ligase, patatin-like phospholipase protein
1.5	0.034–0.073	*deoC*, *mnmG*, PG_RS08805–790	PG1995–92 PG1996	Nucleotide pyrophosphohydrolase, deoxyribose-phosphate aldolase, d-tyrosyl-tRNA(Tyr)deacylase, MnmG
3.2	2.13807E-05	PG_RS08850	PG2006	Hypothetical protein
2.0	9.4623E-05	PG_RS08855	PG2008	TonB-dependent receptor
1.4	0.089	PG_RS09320	PG2102	T9SS type A sorting domain-containing protein
1.4	0.128	PG_RS09460	PG2133	FimB/Mfa2 family fimbrial subunit
1.4	0.032	*ruvX* PG_RS09775	PG2202	Holliday junction resolvase RuvX

a The order of the listed genes is according to the old locus tag. Genes clustering together on the chromosome (potential operons) are shaded in gray. The strain is under NC_002950 (CDS [coding DNA sequence]).

b Ratio of gene expression in bacteria grown under iron-depleted compared with that under iron-rich conditions.

c *P* value from an experiment performed in four independent biological replicates.

**FIG 2:**
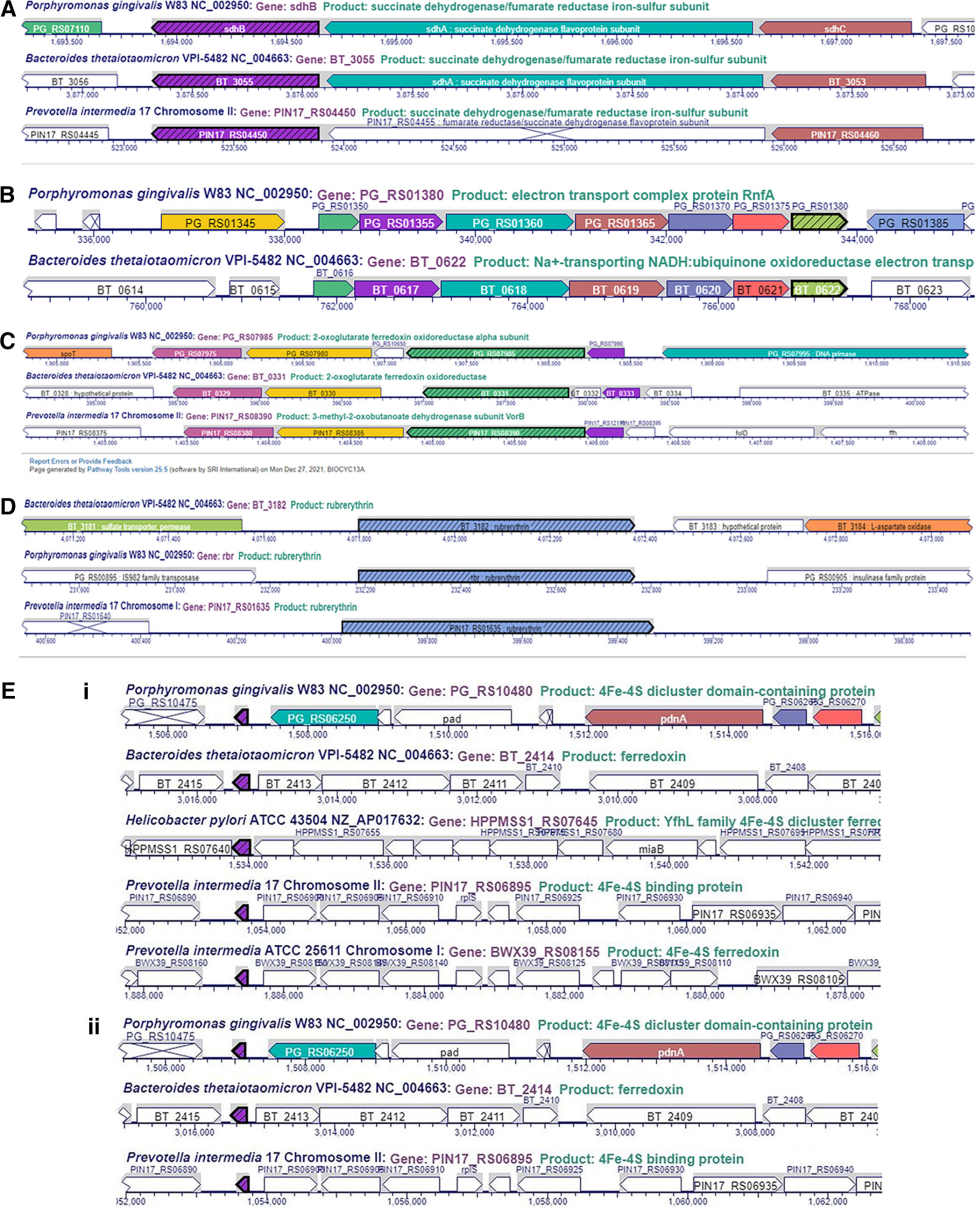
Comparison of loci downregulated in *Bacteroides*. (A) Succinate dehydrogenase/fumarate reductase *sdh* locus in *Bacteroidetes*. Comparison of genomic locus of P. gingivalis W83 (PG_RS07115 to 125, PG1614 to 16), B. thetaiotaomicron VPI-5482 (BT_3053 to 55), and P. intermedia 17 (PIN17_RS04460 to 50). (B) Electron transport *rnf* locus in *Bacteroidetes*. Comparison of genomic locus of P. gingivalis W83 (PG_RS01350 to 80, PG0302 to 308) and B. thetaiotaomicron VPI-5482 (BT_0617 to 22). No ortholog of the locus was found in P. intermedia 17. (C) Ferredoxin- oxidoreductase (*vor*) locus in *Bacteroidetes*. Comparison of genomic locus of P. gingivalis W83 (PG_RS07975 to 90, PG1809013), B. thetaiotaomicron VPI-5482 (BT_0329 to BT_0333), and P. intermedia 17 (PIN17_RS08380 to 95). (D) Rubrerythrin (*rbr*) locus in *Bacteroidetes*. Comparison of genomic locus of P. gingivalis W83 (PG_RS00900, PG0195), B. thetaiotaomicron VPI-5482 (BT_3182), and P. intermedia 17 (PIN17_RS01635). (E) 4Fe-4S dicluster domain-containing protein (frd) locus in *Bacteroidetes* (purple arrow). Comparison of genomic locus of P. gingivalis W83 (PG_RS10480, PG1421), B. thetaiotaomicron VPI-5482 (BT_2414), and P. intermedia 17 (PIN17_RS06895).

Genes that were most downregulated in P. gingivalis W83 grown under iron-depleted conditions compared with those under iron-replete conditions are listed in Table 2. Genomic location of selected genes is shown in Fig. 2. A total of 67 genes were downregulated 2-fold or more (Table 2). The most highly downregulated gene, PG0195, codes for rubrerythrin and is downregulated 46-fold. Other downregulated genes encoded iron-based metabolism proteins. The first one is the operon PG0302 to PG0309 coding for an electron transport Rnf complex. It is downregulated 2- to 3.8-fold, depending on the gene. Also, operon PG1614 to PG1616 coding for the fumarate reductase/succinate dehydrogenase was downregulated by 3.5- to 3.7-fold, depending on the gene. In addition, the operon PG1809 to PG1813 coding for the oxoglutarate oxidoreductase/dehydrogenase system Vor and flanked by a 4Fe-4S binding protein (ferredoxin) was downregulated by 5.2- to 7.4-fold, depending on the gene. Other, strikingly downregulated genes encode hypothetical proteins (PG_RS11335, PG_RS11350, and PG_RS10005; nomenclature according to new locus tag) (30.8-, 9.2-, and 6.3-fold, respectively). No old locus tags were available for those loci (Table 2).

**TABLE 2:** Genes downregulated in P. gingivalis W83 at least 2-fold under iron depleted conditions^*a*^

Fold change^*b*^	*P* value^*c*^	Gene locus_tag	Old_locus_tag	Product
−30.8	0.058	PG_RS11335		Hypothetical protein
−9.2	0.226	PG_RS11350, PG_RS10690		DUF1661 domain-containing protein
−6.3	0.055	PG_RS10005		Hypothetical protein
−5.6	4.9036E-12	PG_RS10650		Hypothetical protein
−3.8	0.082	PG_RS10940, PG_RS11290		DUF1661 domain-containing protein
−3.7	0.491	PG_RS11360		Hypothetical protein
−3.1	0.00203383	PG_RS10460, PG_RS06160		DUF1661 domain-containing protein, transposase
−3.0	0.106	PG_RS11285		Hypothetical protein
−2.9	0.045	PG_RS10395		Hypothetical protein
−2.6	0.227	PG_RS11115		DUF1661 domain-containing protein
−2.6	0.338	PG_RS10535		DUF1661 domain-containing protein
−2.6	0.0003	PG_RS04140		IS*5* family transposase
−2.4	0.252	PG_RS10550		DUF1661 domain-containing protein
−2.3	0.669	PG_RS10870, PG_RS10875		DUF1661 domain-containing protein, hypothetical protein
−2.2	0.302	PG_RS11000		DUF1661 domain-containing protein
−2.1	0.473	PG_RS10580		DUF1661 domain-containing protein
−2.1	0.005	PG_RS02100		DUF4248 domain-containing protein
−2.0	0.035	PG_RS05410		IS*5* family transposase
−2.0	0.115	PG_RS10200		DUF1661 domain-containing protein
−1.9	0.152	PG_RS11050		IS*3* family transposase
−1.9	0.212	PG_RS10250		Glycine cleavage system H protein
−2.0	0.0009	PG_RS00510	PG0110	Glycosyltransferase
−2.5	0.135	PG_RS00810	PG0177	IS*4*-like element IS1598 family transposase
−46.1	0	PG_RS00900	PG0195	Rubrerythrin family protein
−2.2	0.118	PG_RS01030	PG0225	IS*4*-like element IS*1598* family transposase
−3.8 to −2.3	8.9495E-13 to 0.008	PG_RS01350 to 390	PG0302 to 310	RnfABCDGE type electron transport complex
−2.0	0.127	PG_RS11275	PG0442, PG_0442	Hypothetical protein
−4.1	0.195	PG_RS02045	PG0460	IS*5*-like element ISPg8 family transposase
−3.6	0.042	PG_RS10105	PG0524, PG_0524	DUF1661 domain-containing protein
−2.2	0.077	PG_RS10115, PG_RS10120	PG0545, PG_0545, PG0546, PG_0546	Restriction endonuclease subunit S, helix-turn-helix domain-containing protein
−2.1	0.064	PG_RS02500, PG_RS02505	PG0563, PG0564	Hypothetical protein
−4.3	4.4923E-10	PG_RS04540	PG1031	IS*5* family transposase
−2.0	1.5145E-08 to 0.0002	PG_RS05185 to 90	PG1171 to 72	(Fe-S)-binding protein, lactate utilization protein
−2.6	0.044	PG_RS05275	PG1197	IS*5* family transposase
−1.9	0.0007	*nrdG* PG_RS05545	PG1259	Anaerobic ribonucleoside-triphosphate reductase activating protein
−2.0	0.139	PG_RS06340	PG1445	RteC domain-containing protein
−3.1	4.5733E-06	PG_RS06360	PG1448	IS*5* family transposase
−2.1	0.186	PG_RS06425	PG1463	Hypothetical protein
−2.0	0.002	PG_RS06610	PG1500	Helicase
−2.1	0.196	PG_RS10525	PG1527, PG1527	Helix-turn-helix domain-containing protein
−2.0	0.005	PG_RS06820	PG1545	Superoxide dismutase
−1.9	0.056	PG_RS06925	PG1573	Crp/Fnr family transcriptional regulator
−3.7 to −3.5	5.6732E-14 to2.5498E-12	*sdhB,* PG_RS07115 to 25	PG1614 to 16	Succinate dehydrogenase/fumarate reductase iron-sulfur, flavoprotein, cytochrome B subunits
−7.4 to −5.1	0 to 1.8874E-15	PG_RS07975 to 990	PG1809 to 13	Oxidoreductase family proteins, FeS binding protein
−2.8	0.084	PG_RS10710, PG_RS11190	PG1980, PG_1980	DUF1661 domain-containing protein
−3.0	0.013	PG_RS11390	PG2114, PG_2114	Hypothetical protein
−2.0	0.184	PG_RS09375	PG2115	DUF1661 domain-containing protein
−2.5	0.008	PG_RS09740	PG2194	IS*4*-like element IS*1598* family transposase

a The order of the listed genes is according to the old locus tag. Genes clustering together on the chromosome (potential operons) are shaded in gray. The strain is under NC_002950 (CDS [coding DNA sequence]).

b Ratio of gene expression in bacteria grown under iron-depleted compared with that under iron-rich conditions.

c *P* value from an experiment performed in four independent biological replicates.

The conclusion from the above-described regulated genes is that iron/hemin uptake and iron-independent metabolism mechanisms are drastically upregulated while the iron-dependent metabolism and oxidative stress defense mechanisms are downregulated under iron-chelated conditions. These results were also verified using P. gingivalis ATCC33277 (see Table S1 and S2 in the supplemental material). Of note, 3 DUF1661 domain-containing protein-coding genes (upregulated by 9-, 3.7-, 3-, and >2-fold) and 13 DUF1661 domain-containing protein-encoding genes were downregulated. Most of them are genes coding for small proteins specific for P. gingivalis (except one encoded by PG0174 [PG_RS00800]).

### Iron-dependent stimulon of P. intermedia.

**FIG 3:**
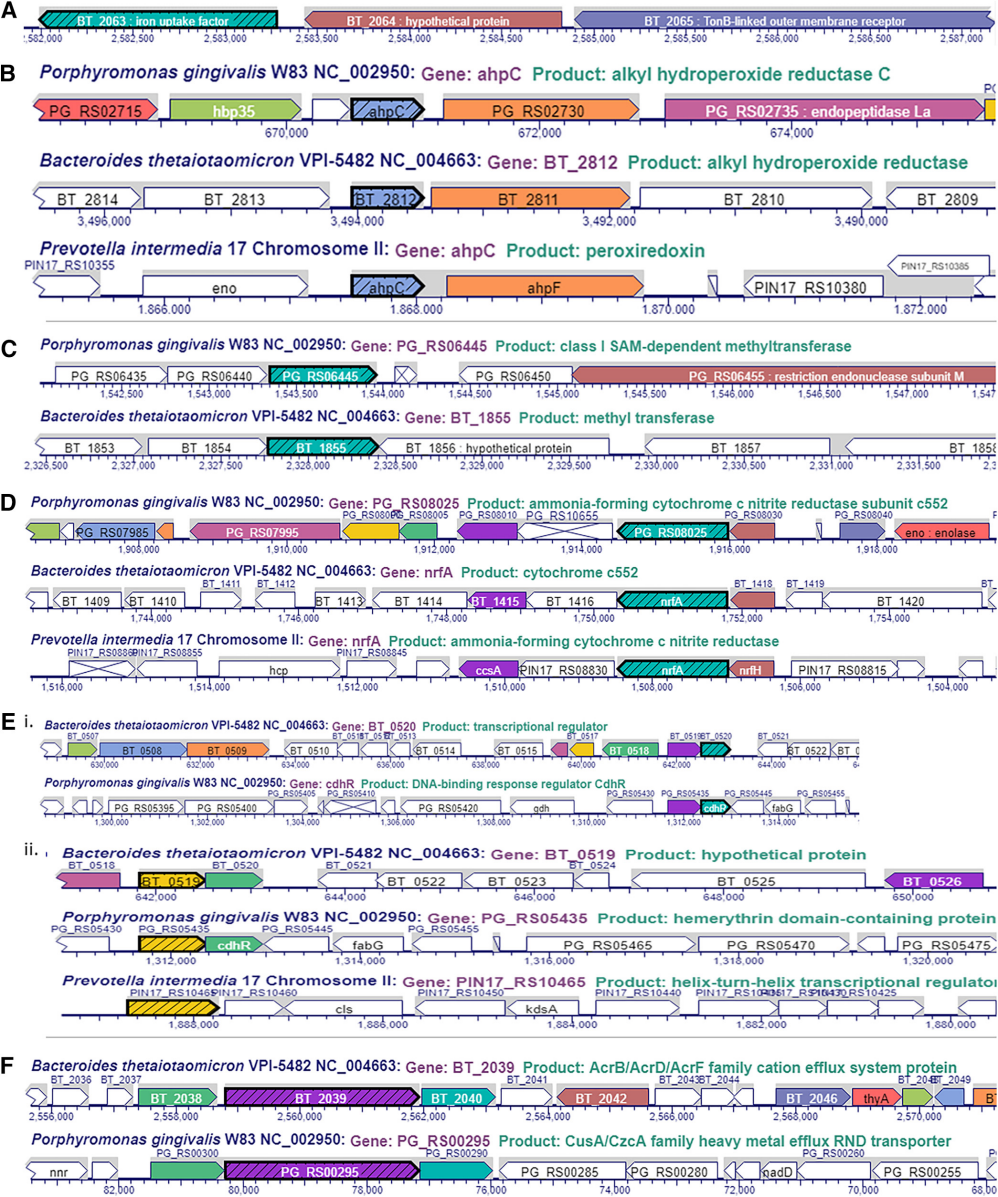

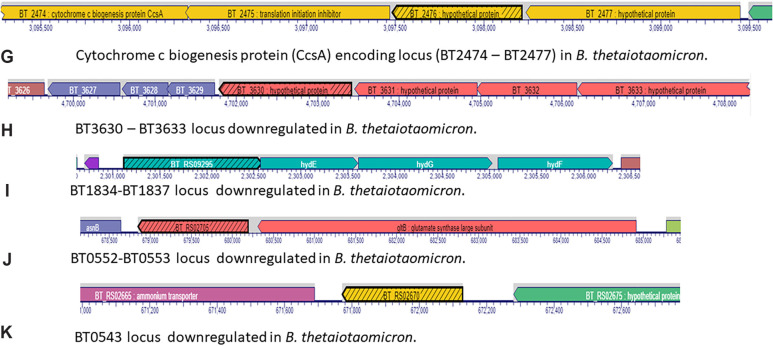
Comparison of other loci regulated by iron in *Bacteroidetes*. (A) Xenosiderophore-mediated iron acquisition encoding a locus in B. thetaiotaomicron (BT_2063 to 2065). The BT_2065 locus coding for the TonB-outer membrane receptor has an ortholog in P. intermedia. (B) *ahpCF* locus in *Bacteroidetes*. Comparison of genomic locus of P. gingivalis W83 (new locus tag, PG_RS02725 to 30; old locus tag, PG0618 to 19), B. thetaiotaomicron VPI-5482 (BT2811 to 12), and P. intermedia 17 (*ahpC-ahpF*). (C) SAM-methyltransferase encoding locus in *Bacteroidetes*. Comparison of genomic locus of P. gingivalis W83 (new locus tag, PG_RS06445; old locus tag, PG1467) and B. thetaiotaomicron VPI-5482 (BT_1855). No similar locus was identified in P. intermedia 17. (D) *nrfAH* locus in *Bacteroidetes*. Comparison of genomic locus of P. gingivalis W83 (new locus tag, PG_RS0825 to 30; old locus tag, PG1820 to 21), B. thetaiotaomicron VPI-5482 (BT_1417 to 18), and P. intermedia 17. (E) Hemerythrin-domain containing regulator (CdhR in P. gingivalis). (i) Identical operon coding for an hemerythrin-domain followed by a DNA-binding regulator (CdhR) is present in P. gingivalis (new locus tag, PG_RS05435 – cdhR; old locus tag, PG1236 to 37) and B. thetaiotaomicron (BT_0519 to 520). (ii) In P. intermedia, the operon is fused, thus producing one regulatory PIN17_RS10465 protein. (F) Cation efflux system present in B. thetaiotaomicron (BT_2038 to 2040) and P. gingivalis (new locus tag, PG_RS00295, PG_RS00300, and PG_RS00290; old locus tag, PG0063 to 65). (G) BT_2063 to 2065. Xenosiderophore-mediated iron acquisition encoding locus in B. thetaiotaomicron. (H) Cytochrome c biogenesis protein (CcsA) encoding locus in B. thetaiotaomicron. (I) BT_3630 to BT_3633 locus in B. thetaiotaomicron. (J) BT1834 to 37 locus downregulated in B. thetaiotaomicron. (K) BT0552 to 53 locus downregulated in B. thetaiotaomicron. (M) BT0543 locus downregulated in B. thetaiotaomicron.

The most drastically regulated genes in P. intermedia OMA14 grown without iron compared with the bacterium grown under iron-replete conditions are listed in Table 3 and 4. The genomic loci of several of those genes are also depicted in Fig. 1 to 3. There were 101 genes upregulated at least 2-fold in P. intermedia OMA14 (Table 3). Among the most upregulated genes were ones encoding metabolic proteins PIOMA14_I_0020 to PIOMA14_I_0021 (*fldA*, flavodoxin) and DUF2023 (family protein) (159- and 164-fold, respectively). Also, there were multiple operons coding for the TetR/AcrR family transcriptional regulator drastically upregulated under low iron conditions. Thus, on the large chromosome, the operon PIOMA14_I_0603 to PIOMA14_I_0605 coding for a TonB-dependent receptor, a hypothetical protein, and a TetR/AcrR family transcriptional regulator (129-, 289-, and 268- fold, respectively) were significantly upregulated. Also, the operon PIOMA14_I_0908 to PIOMA14_I_0914 coded for the TetR/AcrR family transcriptional regulator, two ABC transporter ATP-binding proteins, MMPL family transporter, outer membrane lipoprotein-sorting protein, hypothetical protein, and hypothetical protein, and its site-specific integrase was significantly upregulated (131- to 137-fold, depending on a gene in the operon). Furthermore, the operon PIOMA14_I_1565 to PIOMA_I_1572 encoding a TetR/AcrR family transcriptional regulator, ABC transporter ATP-binding protein, ABC transporter ATP-binding protein, MMPL family transporter, outer membrane lipoprotein-sorting protein, hypothetical protein, hypothetical protein, and site-specific integrase was upregulated by 40-, 81-, 223-, 106-, 76-, 99-, and 54-fold, respectively, depending on the gene. Finally, facing in the opposite direction, an operon with a similar composition as the proteins encoded by the genes in the locus PIOMA14_I_1509 to PIOMA14_I_1516 included TetR/AcrR family transcriptional regulator, ABC transporter ATP-binding protein, ABC transporter ATP-binding protein, MMPL family transporter, outer membrane lipoprotein-sorting protein, hypothetical protein, and hypothetical protein (putative site-specific integrase) and were upregulated by 43-, 96-, 136-, 108-, 48-, 99-, and 48-fold, respectively. Present on the small chromosome was the operon PIOMA14_II_0447 to PIOMA14_II_0449 coding for TetR/AcrR family transcriptional regulator, ABC transporter ATP-binding protein, and ABC transporter ATP-binding protein that were upregulated by 246-, 102-, and 851-fold, respectively.

**TABLE 3:** Genes upregulated at least 2-fold in Prevotella intermedia OMA14 grown under iron-depleted conditions^*a*^

Fold change^*b*^	*P* value^*c*^	Gene locus_tag (genome CDS)	Old_locus_tag (genome CDS)^*d*^	Product (genome CDS)
8.5	2.42E-07	PIOMA14_RS09340		Hypothetical protein
159.3–163.7	0	fldA PIOMA14_RS00100–05	PIOMA14_I_0020–21	Flavodoxin FldA and DUF2023 family protein
3.0	7.83E-05	PIOMA14_RS00245	PIOMA14_I_0051	Helix-turn-helix transcriptional regulator
2.5	0.008996–0.018805	groL PIOMA14_RS01175-80	PIOMA14_I_0239–40	Cochaperone GroES
2.2	0.033994	PIOMA14_RS01655	PIOMA14_I_0334	DnaJ domain-containing protein
34.5	0.030959	PIOMA14_RS01765	PIOMA14_I_0355	IS*982* family transposase
2.4	0.02828	PIOMA14_RS01930	PIOMA14_I_0382	Nucleotide exchange factor GrpE
3.2	0.003992	PIOMA14_RS02095	PIOMA14_I_0415	Hypothetical protein
2.791497	0.041754	PIOMA14_RS02745–50	PIOMA14_I_0549–50	Hypothetical protein, putative porin
128.7–288.6	0	PIOMA14_RS03025–35	PIOMA14_I_0603–5	TonB-dependent transport protein and TetR/AcrR regulator
2.2	0.038591	PIOMA14_RS03345	PIOMA14_I_0663	DUF4359 domain-containing protein
4.6	0.029493	PIOMA14_RS03625	PIOMA14_I_0716	HlyD family secretion protein
2.3–2.4	0–0.003328	PIOMA14_RS03855–70	PIOMA14_I_0759–62	Cof-type HAD-IIB family hydrolase, patatin-like phospholipase and metal transport system
5.2–33.2	0–5.3E-10	PIOMA14_RS04100–120	PIOMA14_I_0804–08	TonB-dependent transport system
64.1	0	PIOMA14_RS04195	PIOMA14_I_0824	Class I SAM-dependent methyltransferase
2.1	0.042773	PIOMA14_RS04425	PIOMA14_I_0867	TlpA family protein disulfide reductase
37.2–131.7	0–6.5E-09	PIOMA14_RS04615–650	PIOMA14_I_0908–15	TetR/AcrR family transcriptional regulator and ABC transport system
12.2	7.41E-12	PIOMA14_RS04770	PIOMA14_I_0939	Isoprenylcysteine carboxyl methyltransferase family protein
3.9	0.033802	PIOMA14_RS05150, PIOMA14_RS05155	PIOMA14_I_1016, PIOMA14_I_1017	Hypothetical protein
2.0–2.7	0.000167–0.029173	PIOMA14_RS05715–20	PIOMA14_I_1122–23	Hypothetical protein
8.5–116.1	0–8.16E-12	hmuY PIOMA14_RS06100–110	PIOMA14_I_1196–98	HmuY family protein and T9SS protein
4.4	4.89E-06	PIOMA14_RS06320	PIOMA14_I_1238	C69 family dipeptidase
70.2	0	PIOMA14_RS06705	PIOMA14_I_1314	TonB-dependent receptor
3.1	9.03E-05	PIOMA14_RS06815	PIOMA14_I_1336	Hypothetical protein
2.1	0.021645	PIOMA14_RS06835	PIOMA14_I_1340	Hypothetical protein
3.1	0.006622	PIOMA14_RS06860	PIOMA14_I_1344	Hypothetical protein
2.2	0.031637	PIOMA14_RS07025	PIOMA14_I_1378	DUF3781 domain-containing protein
2.5	0.026707	PIOMA14_RS07075	PIOMA14_I_1387	Acyl carrier protein
3.9	5.15E-05	PIOMA14_RS07460	PIOMA14_I_1468	Heavy metal translocating P-type ATPase
42.7–136.4	0–2.26E-08	PIOMA14_RS07670–7705	PIOMA14_I_1509–1516	TonB dep transpot system and TetR/AcrR transcriptional regulator
40.46–222.63	0–6.99E-08	PIOMA14_RS07920–55	PIOMA14_I_1565–72	TetR/AcrR family transcriptional regulator and ABC transport system
2.1	0.035693	PIOMA14_RS08015	PIOMA14_I_1586	Hypothetical protein
2.3	0.041384	PIOMA14_RS09030–35	PIOMA14_I_1784–85	Sigma-70 family RNA polymerase sigma factor, hypothetical protein
7.8–11.3	0–1.11E-15	PIOMA14_RS09315–335	PIOMA14_I_1836–40	Hypothetical protein, PorV/PorQ protein, S8 family serine peptidase, BACON domain protein
9.4–72.4	0–6.09E-07	PIOMA14_RS09520–30	PIOMA14_I_1877–79	TonB-dep. receptor, peptidase, 50S protein
2.2	0.030232	PIOMA14_RS09745	PIOMA14_I_1919	Trypsin-like peptidase domain protein
2.0	0.028127	trxA PIOMA14_RS09905	PIOMA14_I_1948	Thioredoxin
2.3	0.000906	PIOMA14_RS10235	PIOMA14_II_0010	Methylaspartate ammonia-lyase
2.2–2.5	0.004026–0.012522	PIOMA14_RS10240–45	PIOMA14_II_0011–12	Acyclic terpene utilization AtuA family protein
3.6	0.022164	PIOMA14_RS14585	PIOMA14_II_0078	Hypothetical protein
2.4–8.6	6.99E-15–0.014783	PIOMA14_RS10720–745	PIOMA14_II_0099–104	YeiH family putative sulfate export transporter
2.1	0.03463	PIOMA14_RS11675	PIOMA14_II_0286	Hypothetical protein
21.6 - 238.6	0–2.01E-05	hmuV – Y, PIOMA14_RS12200	PIOMA14_II_0397	Hmu TonB-dependent hemin uptake
5.7–6.7	1.62E-09–6.44E-09	PIOMA14_RS12410–20	PIOMA14_II_0430–32	TonB transport system
102.3–850.6	0–3.66E-15	PIOMA14_RS12490–500	PIOMA14_II_0447–49	TetR/AcrR regulator and ABC transporter
2.2–2.4	0.045456	PIOMA14_RS12590–95	PIOMA14_II_0466–7	Hypothetical proteins
2.8	0.023653	htpG PIOMA14_RS13475	PIOMA14_II_0665	Molecular chaperone HtpG
2.0	0.010571	PIOMA14_RS13585	PIOMA14_II_0685	Dipeptidase

a The order of the listed genes is according to the old locus tag. Genes clustering together on the chromosome (potential operons) are shaded in gray.

b Ratio range of gene expression in bacteria grown under iron-depleted compared with iron-rich conditions.

c *P* value range of 0.1 from an experiment performed in four independent biological replicates.

d PIOMA14_I designates large chromosome while PIOMA14_II designates small chromosome.

**TABLE 4:** Genes downregulated in P. intermedia OMA14 under iron-depleted conditions^*a*^

Fold change^*b*^	*P* value^*c*^	Gene locus_tag (genome CDS)	Old_locus_tag (genome CDS)^*d*^	Product (genome CDS)
−2.5	0.038	PIOMA14_RS11990		Hypothetical protein
−2.4	0.003	PIOMA14_RS00585	PIOMA14_I_0117	TonB-dependent receptor
−2.2 to −3.4	0.030 to 0.056	PIOMA14_RS00635 to 45, PIOMA14_RS00640	PIOMA14_I_0128 to 31	Hypothetical proteins and *N*-acetylmuramoyl-l-alanina amidase
−3.4	0.041	PIOMA14_RS01695	PIOMA14_I_0342	Hypothetical protein
−2.3	0.008	PIOMA14_RS02580	PIOMA14_I_0518	Porin
−4.3	5.425E-06	*nifJ/pfor* PIOMA14_RS02595	PIOMA14_I_0521	Pyruvate:ferredoxin (flavodoxin) oxidoreductase
−2.9	0.017	PIOMA14_RS02650	PIOMA14_I_0531	Hypothetical protein
−2.6	0.002	PIOMA14_RS02655	PIOMA14_I_0532	Choice-of-anchor J domain-containing protein
−5.3	0.011	PIOMA14_RS02795	PIOMA14_I_0557	IS*982* family transposase
−2.1	0.004	PIOMA14_RS02865	PIOMA14_I_0571	Bifunctional dihydroorotate dehydrogenase B NAD binding subunit/NADPH-dependent glutamate synthase
−2.4	0.035	PIOMA14_RS03010	PIOMA14_I_0600	Hypothetical protein
−3.0 to −2.0	0.00040257	PIOMA14_RS04855 to 60	PIOMA14_I_0955 to 56	YitT family protein and porin
−8.9	0.003	PIOMA14_RS05315	PIOMA14_I_1049	4Fe-4S binding protein
−2.4	0.013	PIOMA14_RS05915	PIOMA14_I_1159	Acyltransferase family protein
−3.2	0.095	PIOMA14_RS06410	PIOMA14_I_1256	Site-specific integrase
−3.2	0.027	PIOMA14_RS06655	PIOMA14_I_1304	GntR family transcriptional regulator
−9.3 to −8.6	2.933E-12 to 1.7193E-11	*fum1* PIOMA14_RS07200 to 210	PIOMA14_I_1410 to 12	Succinate dehydrogenase/fumarate reductase cytochrome b subunit, flavoprotein subunit, Fe-S subunit
−2.6	0.001	PIOMA14_RS08040	PIOMA14_I_1591	Choice-of-anchor J domain-containing protein
−2.1	0.006	PIOMA14_RS08120	PIOMA14_I_1606	Nucleoside recognition domain-containing protein
−4.9 to −2.1	0.00097	PIOMA14_RS08795 to 830	PIOMA14_I_1739 to 46	TonB dep. Transport system with RagB/SusDEF outer membrane proteins
−3.4	0.0001	PIOMA14_RS10155	PIOMA14_I_1992	Fumarate hydratase
−22.3	0	PIOMA14_RS10750	PIOMA14_II_0105	Rubrerythrin
−2.5	0.001	PIOMA14_RS10830	PIOMA14_II_0117	DUF481 domain-containing protein
−2.2	0.031	PIOMA14_RS11290	PIOMA14_II_0201	Aspartate 1-decarboxylase
−12.5	0.100	PIOMA14_RS11515	PIOMA14_II_0247	Helix-turn-helix domain-containing protein
−3.2	0.081	PIOMA14_RS11580, PIOMA14_RS11585	PIOMA14_II_0265, PIOMA14_II_0266	Relaxase/mobilization nuclease domain-containing protein, hypothetical protein
−2.9 to −2.3	0.007 to 0.029	PIOMA14_RS12025 to 35	PIOMA14_II_0361 to 63	Hypothetical and BACON domain-containing proteins
−2.7	0.026	PIOMA14_RS12045	PIOMA14_II_0365	Hypothetical protein
−3.5	0.0002	PIOMA14_RS12290	PIOMA14_II_0411	Choice-of-anchor J domain-containing protein
-2.0	0.008	PIOMA14_RS12435	PIOMA14_II_0435	Restriction endonuclease subunit S
−2.8 to −2.3	0.0002 to 0.044	PIOMA14_RS13230 to 35	PIOMA14_II_0622 to 23	Hypothetical proteins
−2.9 to −2.3	0.010 to 0.085	PIOMA14_RS14000 to 20	PIOMA14_II_0765 to 69	Hypothetical proteins and *N*-acetylmuramoyl-l-alanine amidase

a The order of the listed genes is according to the old locus tag. Genes clustering together on the chromosome (potential operons) are shaded in gray.

b Ratio of gene expression in bacteria grown under iron-depleted compared with iron-rich conditions.

c *P* value from an experiment performed in four independent biological replicates.

d PIOMA14_I designates the large chromosome, while PIOMA14_II designates the small chromosome.

Two hemin uptake loci were significantly upregulated. The locus PIOMA14_I_1196 (PIOMA14_RS06100) coded for the hemin uptake receptor HmuY, followed by T9SS type A sorting domain-containing protein, and ADP-ribosylglycohydrolase family protein that were upregulated 116-, 104-, and 8.5-fold, respectively. A second *hmu* hemin uptake locus located on the small chromosome PIOMA14_II_0397 to PIOMA14_II_0402 encoded the HmuY family protein, TonB-dependent receptor, cobaltochelatase subunit CobN, hypothetical protein, MotA/TolQ/ExbB proton channel family protein, and DUF2149 domain-containing protein that were upregulated 239-, 140-, 127-, 115-, 60-, and 22-fold respectively. This *P. intermedia hmu* operon bears significant similarity with the P. gingivalis *hmu* operon identified by Lewis et al. (14). Of interest, among the upregulated genes is also locus (PIOMA14_II_0099 to PIOMA14_II_104) coding for sulfate transporter, iron transport protein (FeoB), and anaerobic ribonucleotide-triphosphate reductase (NrdD and NrdG).

Downregulated by at least 2-fold was 49 genes (Table 4). PIOMA14_II_0105 coding for rubrerythrin was the most drastically downregulated gene (22.3-fold). Also significantly downregulated was operon PIOMA14_I_1410 to PIOMA14_I_1412 encoding the fumarate reductase/succinate dehydrogenase system (8.5-, 9.2-, and 9.3-fold, respectively). PIOMA14_I_1049 (PIOMA14_RS05315) coding for the 4Fe-4S binding protein (ferredoxin, similar to PG1421 and BT2414) was also downregulated by 8.9-fold (Fig. 2E; see Fig. S1 in the supplemental material). The major iron-dependent metabolic enzyme pyruvate:ferredoxin (flavodoxin) oxidoreductase, PIOMA14_I_0521, was downregulated by 4.3-fold. Finally, PIOMA14_II_0247 coding for the helix-turn-helix domain-containing protein was downregulated by 12.5-fold. Many other genes coding for a transport system, such as the SusD/E system (PIOMA14_I_1739 to PIOMA14_I_1746), TonB-dependent system (PIOMA14_I_0117), porin (PIOMA14_I_0518 and PIOMA14_I_0956), and hypothetical proteins, were downregulated (Table 4).

In summary, in P. intermedia, iron depletion leads to overexpression of iron-independent metabolic mechanisms and iron uptake mechanisms and downregulation of iron-based metabolism and oxidative stress response mechanisms.

### Iron-dependent stimulon of B. thetaiotaomicron.

Genes regulated by iron levels in B. thetaiotaomicron VPI BT5482 Δ*tdk* are listed in Table 5 and 6. Genomic loci of selected genes are also depicted in Fig. 1 to 3. We identified 323 genes upregulated by at least 2-fold (Table 5). The most drastically regulated one was a locus composed of three genes, namely, BT2063 to BT2065 (upregulated by 489.4-, 676.1-, and 663-fold, respectively, depending on the gene). The locus codes for a TonB-dependent receptor and two proteins with the DUF4374 domain and with the PepSY domain, respectively (2). This locus was shown recently to be involved in xenosiderophore utilization and highly upregulated in the colitis model (2). It is noteworthy that the same bacterial strain has been used in the colitis model study and in our analyses, thus enabling us to directly compare the data. In addition, operon BT0491 to BT0498 was highly upregulated and encoded the Hmu-like hemin uptake system (upregulation ranging from 108.9- to 290.8-fold, depending on the gene). BT0507 to BT0509 coding for the TetR/AcrR family transcriptional regulator and two ABC transporter ATP-binding proteins were upregulated by 51.2-, 516.2-, and 675.5-fold, respectively. Highly upregulated was the locus BT2473 to BT2482 encoding fimbrillin-family protein, two cytochrome c biogenesis proteins (CcsA), porin, thiol oxidoreductase, peptidase M75, helix-turn-helix transcriptional regulator, and three hypothetical proteins with 69.4- to 323.7-fold upregulation depending on the gene. It is also noteworthy that BT2479 was also upregulated in the colitis model (2). The locus BT3625 to BT3633 coding for proteins with a PepSY-associated TM helix domain, DUF4857 domain, fimbrillin protein, TonB-dependent receptor, ABC transporter ATP-binding protein, and hypothetical protein was upregulated by 36.7- to 128-fold, depending on the gene within the locus.

**TABLE 5:** Genes upregulated at least 2-fold in B. thetaiotaomicron BT5482 Δ*tdk* under iron-depleted conditions^*a*^

Fold change^*b*^	*P* value^*c*^	Gene locus tag	Old_locus_tag	Product
2.155	0.003	BT_RS06005		Helix-turn-helix domain-containing protein
2.269	0.147	BT_RS09290		Hypothetical protein
2.546	0.199	BT_RS09765		Site-specific integrase
3.226	0.097	BT_RS12005		Hypothetical protein
3.551	0.437	BT_RS05690		DUF3408 domain-containing protein
3.551	0.437	BT_RS05715		Hypothetical protein
3.551	0.437	BT_RS13325		Tyrosine-type recombinase/integrase
3.551	0.437	BT_RS20280		Helix-turn-helix domain-containing protein
3.551	0.437	BT_RS12405		Helix-turn-helix domain-containing protein
5.921	0.25	BT_RS13060		Hypothetical protein
5.968	0.248	BT_RS05060		AAA family ATPase
5.968	0.248	BT_RS05725		Hypothetical protein
7.034	0.053	BT_RS12380		Hypothetical protein
8.291	0.161	BT_RS05905		HU family DNA-binding protein
17.77	0.049	BT_RS00115		Transposase
42.96	0.01	BT_RS12570, BT_RS12575		Abi family protein
54.98	0.007	BT_RS24385		Hypothetical protein
150.9	0	BT_RS02430		Cobaltochelatase subunit CobN
2.207	0.272	BT_RS00045, BT_RS00050	BT0010–11	Hypothetical protein
2.614	0.232	BT_RS00060	BT0013	Hypothetical protein
8.291	0.161	BT_RS00075	BT0016	RteC domain-containing protein
3.551	0.437	BT_RS00090	BT0019	Hypothetical protein
15.4	0.062	BT_RS00120	BT0028	Alkaline phosphatase
3.201	0.021	BT_RS00360	BT0076	Site-specific integrase
5.921	0.25	BT_RS00370	BT0078	JAB domain-containing protein
3.551	0.437	BT_RS00485, BT_RS00490	BT0100–101	Hypothetical protein, relaxase/mobilization nuclease domain-containing protein
3.551	0.437	BT_RS00525, BT_RS00530	BT0108–09	AAA family ATPase, helix-turn-helix domain protein
2.485	0.089	BT_RS00550	BT0113	Hypothetical protein
5.146	0.071	BT_RS00640	BT0130	SDR family oxidoreductase
2.506	0.28	BT_RS00860	BT0175	TIGR03987 family protein
5.968	0.248	BT_RS00965	BT0196	MFS transporter
2.672	8E-05	BT_RS01060	BT0215	Transcriptional repressor
8.1–3.6	1E-14–0.437	BT_RS01100–1135	BT0224–31	Hypothetical proteins and helix-turn-helix protein
2.2	3E-04	BT_RS01175	BT0238	Anaerobic sulfatase-maturation protein
2.1–2.7	2E-05–0.001	BT_RS01210–240	BT0245–51	AAA family ATPase, metallophosphoesterase, membrane protein, sigma-70 RNA polymerase sigma factor, dihydrooratase
2.2–2.9	6E-05–4E-09	BT_RS01275–80	BT0258–59	Glucosamine-6-phosphate deaminase, aminohydrolyse
5.922	0.25	BT_RS01295	BT0263	Six-hairpin glycosidase
3.089	0.14	BT_RS01435	BT0292	YjbH domain-containing protein
2.29	0.097	BT_RS01480	BT0302	Hypothetical protein
2.138	0.336	BT_RS01655	BT0339	Alpha-xylosidase
2.055	0.009	BT_RS01830	BT0375	Tyrosine-type DNA invertase cluster 3b
3.137	0.02	BT_RS01970	BT0404	DUF3987 domain-containing protein
11.1–290.8	0–4E-08	BT_RS02415–450	BT0491–99	Hmu hemin transport system
2.4–675.5	0–2E-04	BT_RS02470–2505	BT0503–09	TetR/AcrR family transcriptional regulator, TonB-dep receptor, ABC transporter
2.316	0.068	BT_RS02520	BT0512	Hypothetical protein
37.1–598.4	0	BT_RS02535–45 (fldA)	BT0515–17	Quinol oxidase, DUF2023 protein, FldA
16.3–20.0	0–7E-13	BT_RS02555–60	BT0519–20	Hemerythrin domain-containing protein, response regulator transcription factor
2.015	5E-04	BT_RS02575	BT0523	Sugar transferase
2.143	9E-06	BT_RS02580	BT0524	Response regulator
2.173	0.079	BT_RS02600	BT0528	Phosphoribosylanthranilate isomerase
2.0–3.0	2E-06–0.104	BT_RS02615–25, trp	BT0531–33	Aminodeoxychorismate/anthranilate synthase compon. II, anthranilate synthase component I protein, tryptophan synthase beta subunit
2.456	0.088	BT_RS02685	BT0548	Diaminopimelate epimerase
2.105	3E-05	BT_RS02830	BT0577	LysM peptidoglycan-binding domain-containing protein
27.23	0.025	BT_RS02870	BT0585	Flavin reductase
2.2–3.3	5E-07–5E-04	BT_RS03105–115, xth	BT0629–31	Nramp family divalent metal transporter, exodeoxyribonuclease III, C-GCAxxG-C-C family protein
3.551	0.437	BT_RS03285	BT0663	Hypothetical protein
4.354	0.113	lepB	BT0667	Signal peptidase I
4.354	0.119	BT_RS03315	BT0669	Efflux RND transporter periplasmic adaptor subunit
2.7–3.6	8E-05–0.437	BT_RS03630–35	BT0726–27	RtcB family protein, hypothetical protein
3.567	0.02	BT_RS03955	BT0791	NUDIX hydrolase
3.551	0.437	BT_RS04255	BT0851	Hypothetical protein
2.627	0.007	BT_RS04400	BT0878	ATP-binding cassette domain-containing protein
2.0–3.4	7E-08	htpG, BT_RS04515	BT0897–98	Molecular chaperone HtpG
2.0–5.0	8E-05–0.074	BT_RS04640	BT0922	PepSY-like domain-containing proteins, Pas-domain sensor kinase
2.443	3E-04	BT_RS05110–115	BT1015–16	SDR family oxidoreductase, patatin-like phospholipase family protein
3.562	0.178	BT_RS05170	BT1028	RagB/SusD family nutrient uptake outer memb. protein
2.5–8.3	0.002–0.279	BT_RS05245–55	BT1043–45	SusD/RagB family nutrient-binding outer memb.lipoprotein, endoglycosidase, DUF1735 domain protein
3.551	0.437	BT_RS05365	BT1068	Hypothetical protein
3.551	0.437	BT_RS05505	BT1095	Helix-turn-helix domain-containing protein
2.09	0.03	BT_RS05630	BT1116	DUF296 domain-containing protein
2.039	0.049	BT_RS05655	BT1121	Carboxymuconolactone decarboxylase family protein
3.551	0.437	BT_RS05705–10	BT1131	Hypothetical protein
3.1–3.5	2E-10–4E-07	BT_RS05740–50	BT1138–39	Hypothetical protein, site-specific integrase, TonB-dep protein
2.178	0.005	BT_RS05875	BT1163	TolC family protein
2.515	0.214	BT_RS05925	BT1173	DUF4373 domain-containing protein
2.1–2.3	0.032–0.271	BT_RS06120–30	BT1212–14	TolC family protein, efflux RND transporter, ABC transporter
2.933	0.032	BT_RS06235	BT1232	Hypothetical protein
2.017	0.087	BT_RS06340–45	BT1255–56	Hypothetical protein, DUF2089 family protein
2.66	0.219	BT_RS06370	BT1262	Helix-turn-helix transcriptional regulator
2.622	4E-05	BT_RS06380	BT1264	Response regulator
3.566	0.017	BT_RS07020	BT1385	AraC family transcriptional regulator
3.565	0.056	BT_RS07100	BT1401	MFS transporter
3.565	0.09	BT_RS07210	BT1423	ASCH domain-containing protein
3.551	0.437	BT_RS07225	BT1426	DUF1062 domain-containing protein
1.985	0.003	trxA	BT1456	Thioredoxin
2.048	0.067	BT_RS07380–85	BT1457–59	Response regulator transcription factor, histidine kinase
2.088	8E-04	BT_RS07480	BT1477	Fucose isomerase
2.2–3.0	5E-06	BT_RS07965	BT1575–77	2-Hydroxyacid dehydrogenase, pirin family protein, pyridoxamine 5′-phosphate oxidase
3.241	0.002	BT_RS08050	BT1590	Hypothetical protein
5.921	0.25	BT_RS08070, feoB	BT1592	Hypothetical protein, ferrous iron transport protein B
2.9	5E-08	BT_RS08100, BT_RS08105	BT1597–98	Tetratricopeptide repeat protein, HAMP domain-containing histidine kinase
2.3	0.007	BT_RS08130, ccsA	BT1604–5	Cytochrome c biogenesis protein ResB, cytochrome c biogenesis protein CcsA
2.3	0.05	BT_RS08205	BT1618	FecR family protein
15.4	0.062	BT_RS08300	BT1637	Hypothetical protein
15.4–23.9	0	BT_RS08405 (fsa), BT_RS08410, BT_RS0415 (gpmA)	BT1658–60	Fructose-6-phosphate aldolase, 2, 3-diphosphoglycerate-depen. phosphoglycerate mutase
3.6	0.179–0.437	BT_RS08685–95	BT1713–15	HAD-IIIA family hydrolase, sialic acid synthase, glycosyltransferase family protein
2–3.6	0.338–0.437	aepX, BT_RS08725	BT1720–21	Phosphoenolpyruvate mutase, phosphocholine cytidylytransferase family protein
3.6	0.437	BT_RS09045–50	BT1785–86	Hypothetical protein, DUF4270 family protein
2.2–3.1	0.249	BT_RS09090–95	BT1794–95	Response regulator transcription factor, histidine kinase
2.1	0.004	BT_RS09105	BT1797	Helix-turn-helix transcriptional regulator
2.1–2.3	3E-04	groL - BT_RS09270	BT1829–30	Chaperonin GroEL, cochaperone GroES
2.8	0.297	BT_RS09460	BT1864	Class I SAM-dependent methyltransferase
27.0	0.039	BT_RS09485	BT1869	Transposase
2.2	0.125	BT_RS09520	BT1876	DUF4974 domain-containing protein
4.4–6.4	7E-04–0.011	BT_RS09595–605	BT1890–92	UvrD-helicase domain-containing protein, hypothetical protein, IS*3* family transposase
2.1–3.1	7E-04	BT_RS09620	BT1895–97	Hypothetical protein, leucine-rich repeat protein, LemA family protein
3.5–7.4	0–0.009	BT_RS09805–35	BT1933–39	IS*256* family transposase, hypothetical proteins, HEAT repeat domain-containing protein, DUF4876 domain-containing protein, TonB-dependent receptor
3.6	0.437	BT_RS09850	BT1942	Hypothetical protein
2.1	0.342	BT_RS09855–60	BT1944–45	Helix-turn-helix domain-containing protein, ParA family protein
2.7–3.6	0.288–0.437	BT_RS09875–85	BT1948–49	Hypothetical protein
3.6	0.437	BT_RS09950	BT1964	TetR/AcrR family transcriptional regulator
3.6	0.178	BT_RS10035–4040	BT1981–82	Hypothetical protein
2.0	0.007	rpiA	BT1986	Ribose 5-phosphate isomerase A
2.0	0.083	BT_RS10245	BT2024	Hypothetical protein
2.5–3.3	1E-08–1E-05	BT_RS10325–35	BT2038 40	Efflux RND transporter periplasmic adaptor subunit, CusA/CzcA family heavy metal efflux RND transporter, TolC family protein
489.4–676.1	0	BT_RS10455–65	BT2063–65	PepSY domain-containing protein, DUF4374 domain-containing protein, TonB-dependent receptor
2.2	0.009	BT_RS10795–800	BT2132–33	Hypothetical protein, LPS-assembly protein LptD
16.5	0.077	BT_RS10825	BT2138	Transposase
2.0	0.01	BT_RS11045	BT2182	DUF1349 domain-containing protein
3.6	0.437	BT_RS11055–60	BT2184–85	RNA polymerase sigma factor, hypothetical protein
3.6	0.437	BT_RS11235	BT2221	DUF3244 domain-containing protein
2.1	0.209	BT_RS11255	BT2225	DsbA family protein
3.6	0.437	BT_RS11545	BT2283	Hypothetical protein
6.0	0.248	BT_RS11560	BT2286	DUF3873 domain-containing protein
2.8	0.297	BT_RS11570	BT2288	Conjugal transfer protein TraO
2.0	0.129	ltrA	BT2297	Group II intron reverse transcriptase/maturase
3.6	0.437	traG, BT_RS11625	BT2298–99	TraG family conjugative transposon ATPase, DUF4133 domain-containing protein
8.3	0.161	BT_RS11645	BT2303	ParA family protein
3.6	0.437	BT_RS11695, BT_RS11700	BT2312–13	Hypothetical protein, RNA-directed DNA polymerase
3.551	0.437	BT_RS11695, BT_RS11700	BT2312, BT_2312 BT2313, BT_2313	Hypothetical protein, RNA-directed DNA polymerase
6.0	0.25	BT_RS11750	BT2323	DUF3945 domain-containing protein
5.6	0.006	BT_RS11770–75	BT2327–28	Hypothetical protein, DUF3800 domain-containing protein
2.5	0.323	BT_RS11850	BT2345	CPBP family intramembrane metalloprotease
5.9	0.25	BT_RS11880, tnpB	BT2350–51	IS*66* family insertion sequence hypothetical protein, IS*66* family insertion sequence element accessory protein TnpB
2.8	0.003	BT_RS11910	BT2356	Helix-turn-helix transcriptional regulator
3.3	0.008	BT_RS11975	BT2370	DinB family protein
2.0	0.09	BT_RS12065	BT2387	*O*-acetylhomoserine aminocarboxypropyltransferase/cysteine synthase
4.0	0.097	BT_RS12120–25	BT2398–99	Hypothetical protein, IS*3* family transposase
3.6	0.437	BT_RS12255	BT2423	K(+)-transporting ATPase subunit C
3.0	0.147	kdpA	BT2425	Potassium-transporting ATPase subunit KdpA
20.1	0.041	BT_RS12300–05	BT2427–28	IS*3* family transposase, hypothetical protein
3.5	5E-04	BT_RS12410	BT2450	Hypothetical protein
3.1	0.159	BT_RS12425	BT2453	Hypothetical protein
5.9	0.25	BT_RS12430	BT2455	Reverse transcriptase
3.6	0.437	BT_RS12500	BT2468	Helix-turn-helix domain-containing protein
62.7–323.7	0	BT_RS12520–65 (ccsA)	BT2473–82	Fimbrillin family protein, cytochrome c biogenesis protein CcsA, hypothetical proteins, porin, thiol oxidoreductase, peptidase M75, helix-turn-helix transcriptional regulator
2.0–2.8	3E-05–0.006	BT_RS12620–35	BT2494–97	ABC transporter ATP-binding protein, ABC transporter permease, efflux RND transporter periplasmic adaptor subunit
3.2	2E-04	msrB	BT2499	Peptide-methionine (R)-S-oxide reductase MsrB
2.2–9.6	2E-16–0.03	BT_RS12705–10 (cadA)	BT2511–12	Transcriptional regulator, cadmium-translocating P-type ATPase
3.3	0.024	BT_RS12820	BT2535	Hypothetical protein
2.2	4E-04	BT_RS12845	BT2540	Two-component sensor histidine kinase
29.7	0.034	BT_RS12925	BT2556	Transposase
4.4	0.119	BT_RS13010	BT2572	Two pore domain potassium channel family protein
3.2	0.069	BT_RS13030–35	BT2575–76	Hypothetical protein, site-specific integrase
8.3	0.161	traM, BT_RS13140, traK	BT2596, BT_2596 BT2597, BT_2597–98	Conjugative transposon protein TraM, DUF3989 domain protein, conjugative transposon protein TraK
5.9	0.25	BT_RS13320	BT2634	Tyrosine-type recombinase/integrase
3.6	0.437	BT_RS13360	BT2645	PRTRC system protein E
2.1	0.342	BT_RS13370	BT2647	Hypothetical protein
11.5	0.007	BT_RS13510-15	BT2673–74	Hypothetical protein, IS3 family transposase
2.0	2E-04	BT_RS13630	BT2697	Hypothetical protein
2.3	0.001	BT_RS14075	BT2778	Sigma-70 family RNA polymerase sigma factor
2.2	0.249	BT_RS14160	BT2794	HlyD family secretion protein
2.4–8.0	6E-05–0.005	ahpF–ahpF	BT2811, BT_2811	Alkyl hydroperoxide reductase subunit F, peroxiredoxin
18.1–4.5	4E-10–0.049	BT_RS14290–225	BT2818–25	TonB-dependent receptor, RagB/SusD family nutrient uptake outer membrane proteins, SusC/RagA family TonB-linked outer membrane protein, BACON domain-containing proteins, chitinase
3.6	0.437	BT_RS14520	BT2863	Polysaccharide biosynthesis/export family protein
3.6	0.437	BT_RS14585–910	BT2876–81	Glycosyltransferase, acyltransferase, lipopolysaccharide biosynthesis protein, CDP-glycerol glycerophosphotransferase family protein, NAD-dependent epimerase/dehydratase family protein, 2-*C*-methyl-d-erythritol 4-phosphate cytidylyltransferase,
3.6	0.437	BT_RS14645	BT2888	Undecaprenyl/decaprenyl-phosphate alpha-*N*-acetylglucosaminyl 1-phosphate transferase
3.6	0.437	BT_RS14980	BT2954	Hypothetical protein
2.6	0.232	BT_RS15070–75	BT2974–75	Hypothetical protein, NAD-dependent deacylase
2.6	0.036	BT_RS15160	BT2990	Tyrosine-type recombinase/integrase
3.1	0.153	BT_RS15535	BT3064	DUF4248 domain-containing protein
5.9	0.25	BT_RS16085	BT3177	Sulfatase
4.6	0.021	BT_RS16185–90	BT3197–98	Hypothetical protein, DUF4373 domain-containing protein
2.9	0.027	BT_RS17260–65	BT3410	Heavy metal-binding domain-containing protein, hypothetical protein
4.2	0.021	BT_RS17295	BT3416	IS*As1*-like element ISBthe4 family transposase
2.3	0.103	BT_RS17400	BT3438	Hypothetical protein
16.5	0.076	BT_RS17600	BT3480	Transposase
2.5	0.041	BT_RS18045	BT3575	Hypothetical protein
2.6	0.35	BT_RS18235	BT3614	Oxidoreductase
38.1–128.1	0	BT_RS18290–325	BT3625–33	DUF4857 domain-containing protein, hypothetical proteins, ABC transporter ATP-binding protein, PepSY-associated TM helix domain-containing protein, hypothetical protein, DUF4876 domain-containing proteins, TonB-dependent receptor
5.2	0.084	BT_RS18570	BT3682	DUF4361 domain-containing protein
16.5	0.076	BT_RS18690	BT3707	Transposase
3.6	0.437	BT_RS19185–90	BT3805	Response regulator, hypothetical protein
16.5	0.076	BT_RS19290	BT3826	Transposase
2.3–5.0	4E-07–0.006	BT_RS20020–40	BT3968–72	Efflux RND transporter periplasmic adaptor subunit, CusA/CzcA family heavy metal efflux RND transporter, glutathione peroxidase, NUDIX domain-containing protein
3.6	0.437	BT_RS20295	BT4021	Site-specific integrase
5.9	0.25	BT_RS20335	BT4029	Hypothetical protein
3.6	0.437	BT_RS20550	BT4071	Hypothetical protein
2.1	0.02	BT_RS20730	BT4107	PTS sugar transporter subunit IIC
7.5	0.031	BT_RS20790	BT4119	Pectate lyase
3.6	0.437	BT_RS20855	BT4132	Discoidin domain-containing protein
2.0	0.004	BT_RS21325	BT4227	Mfa1 fimbrilin C-terminal domain protein
2.2–3.3	7E-04	BT_RS21360	BT4233–36	DUF3868 domain-containing protein, DUF3575 domain-containing protein, response regulator
6.0	0.248	BT_RS21575–80	BT4274	DUF5030 domain-containing protein, hypothetical protein
2.8	0.297	BT_RS21590, tnpB	BT4276–77	IS*66* family insertion sequence hypothetical protein, accessory protein TnpB
2.7	0.034	BT_RS21980	BT4355	RNA polymerase sigma-70 factor
2.4	6E-05	BT_RS22275	BT4416	Copper homeostasis protein CutC
3.6	0.437	BT_RS22400	BT4441	Leucine-rich repeat protein
2.4	0.015	BT_RS22590	BT4479	Site-specific integrase
2.1	7E-04	BT_RS22655	BT4492	Hypothetical protein
2.3–3.6	0.437	BT_RS22685–30	BT4498–49	Hypothetical protein, site-specific integrase
2.2	0.008	BT_RS22945	BT4552	Response regulator
2.0	7E-04	BT_RS22965	BT4556	NfeD family protein
3.4	0.002	BT_RS23025	BT4567	Histidine kinase
2.0–7.7	0.144	BT_RS23040–45, eno	BT4570–72	Hypothetical protein, RNA polymerase sigma factor, phosphopyruvate hydratase
3.2	0.097	BT_RS23075–80	BT4577–78	Hypothetical protein, IS*3* family transposase
2.0	0.011	BT_RS23250	BT4613	Hypothetical protein
2.0–6.0	4E-04	dnaK, BT_RS23265–85	BT4615 19	Molecular chaperone DnaK, helix-turn-helix domain-containing proteins, site-specific integrase, VirE protein, DUF3987 domain-containing protein
2.1–3.6	0.099–0.359	BT_RS23290–300	BT4621–23	MobC family plasmid mobilization relaxosome protein, relaxase/mobilization nuclease domain-containing, protein, hypothetical protein
2.7	0.012	BT_RS23395–400	BT4643–44	RNA polymerase sigma-70 factor, FecR domain-containing protein
3.4	0.152	BT_RS23610	BT4685	RagB/SusD family nutrient uptake outer memb. protein
5.8	4E-05	BT_RS23755	BT4715	DNA starvation/stationary phase protection protein
2.6–8.3	0–0.043	BT_RS23855–860, pflA, pflB	BT4735–38	DsbA family protein, hypothetical proteins, N-acetylmuramoyl-L-alanine amidase, pyruvate formate lyase-activating protein, formate C-acetyltransferase
2.2	0.259	BT_RS23885	BT4740	IS*110* family transposase
2.1	0.343	BT_RS23930	BT4749	N-6 DNA methylase
6.0	0.248	BT_RS24005	BT4763	DUF3408 domain-containing protein

a The order of the listed genes is according to the old locus tag. Genes clustering together on the chromosome (potential operons) are shaded in gray. The strain is under NC_004663 (CDS).

b Ratio of gene expression in bacteria grown under iron-depleted compared with iron-rich conditions.

c *P* value from an experiment performed in four independent biological replicates.

**TABLE 6:** Genes downregulated at least 2.5-fold in B. thetaiotaomicron BT5482 Δ*tdk* under iron-depleted conditions^*a*^

Fold change^*b*^	*P* value^*c*^	Locus_tag	Old_locus_tag	Product
−9.3	0.132	BT_RS16240		Hypothetical protein
−4.5	1E-04			
−4.2	0.353	BT_RS21305		Hypothetical protein
−4.2	0.353	BT_RS24330		Hypothetical protein
−4.2	0.117	BT_RS00315		Hypothetical protein
−3.2	0.14	BT_RS22770		Helix-turn-helix transcriptional regulator
−3.1	0.235	BT_RS11535		Hypothetical protein
−3.1	0.235	BT_RS17910		PLDc N-terminal domain-containing protein
−2.5	0.57	BT_RS24325		Hypothetical protein
−2.5	0.57			
−2.5	0.57	BT_RS05605		Hypothetical protein
−2.5	0.57	BT_RS10270		DUF4373 domain-containing protein
−2.5	0.57	BT_RS12485		Hypothetical protein
−2.5	0.57	BT_RS05530		Transposase
−2.5	0.57	BT_RS09845		Hypothetical protein
−2.5	0.57	BT_RS13180		DUF4134 domain-containing protein
−2.5	0.57			
−2.5	0.57	BT_RS12025		Acyltransferase
−2.5	0.57	BT_RS04725		Hypothetical protein
−2.5	0.57	BT_RS08645		Smalltalk protein
−2.5	0.57	BT_RS15170		DUF3853 family protein
−2.5	0.57	BT_RS24395		Hypothetical protein
−2.5	0.57	BT_RS21300		Hypothetical protein
−2.5	0.326	BT_RS05520		Helix-turn-helix domain-containing protein
−2.8	0.109	BT_RS03120		Winged helix-turn-helix domain protein
−8.1	0.169	BT_RS00010	BT0002	Hypothetical protein
−4.2	0.353	BT_RS00020	BT0004	Hypothetical protein
−5.9	0.241	BT_RS00105	BT0022	Helix-turn-helix domain-containing protein
−2.5	0.042	BT_RS00190, BT_RS00195	BT0044	Glycosyltransferase family 2 protein, acyltransferase
−3.1	0.003	BT_RS00270	BT0060	Polysaccharide biosynthesis/export family protein
−4.4 to −2.9	2E-06 to 0.005	BT_RS00275 to 80	BT0061 to 62	Polysaccharide biosynthesis tyrosine autokinase, hypothetical protein
−3.49	0.044	BT_RS00305	BT0067	Hypothetical protein
−4.2 to −2.5	0.353 to 0.57	traNMK, BT_RS00410 to 425	BT0086 to 89	Conjugative transposon proteins tranmk
−4.2	0.352	BT_RS00435	BT0091, BT_0091	DUF4141 domain-containing protein
−2.5	0.57	traG, BT_RS00445 to 50	BT0093 to 94	TraG conjugative transposon ATPase, DUF4133 domain- protein
−2.5	0.57	BT_RS00460	BT0096	Hypothetical protein
−4.2	0.353	BT_RS00470, BT_RS00475	BT0098	AAA family ATPase, hypothetical protein
−4.2	0.352	BT_RS00500 to 05	BT0103 to 04	DUF3945 domain-containing protein, hypothetical protein
−4.2 to −2.5	0.353 to 0.57	BT_RS00535 to 40	BT0110 to 11	RteC domain-containing protein, hypothetical protein
−4.1	0.177	BT_RS00560	BT0115	Arsenate reductase ArsC
−5.4	5E-09	BT_RS00610	BT0124	2Fe-2S iron-sulfur cluster binding protein
−4.2	0.353	BT_RS00645, BT_RS00650	BT0131	IS*4* family transposase
−12.8 to −11	0	BT_RS00850 to 55	BT0173 to 74	GGGtGRT protein, nitrogen-fixing protein NifU
−2.7	0.038	BT_RS00935	BT0190	TonB-dependent receptor
−12.7 to −2.5	0.084 to 0.57	BT_RS01020 to 25	BT0207 to 08	RagB/SusD family nutrient uptake outer membrane protein and DUF4984 domain protein
−5.9 to −2.6	0.152 to 0.241	BT_RS01040 to 50	BT0211 to 13	BACON domain-containing protein, S8 family serine peptidase, DUF1573 domain protein
−29.5 to −2.6	0.007 to 0.071	BT_RS01325 to 355	BT0269 to 75	RagB/SusD family nutrient uptake outer membrane protein, hypothetical protein, DUF5007 domain-containing protein, RagB/SusD family nutrient uptake outer membrane protein, DUF5124 domain-containing protein
−7.6 to −2.6	0.033 to 0.18	BT_RS01365 to 75	BT0277 to 79	Carbohydrate-binding protein, alkaline phosphatase family protein, hypothetical protein
−3.1	0.227	BT_RS01440	BT0293	YjbH domain-containing protein
−2.9	0.003	BT_RS01470	BT0299	efflux RND transporter permease subunit
−3.5 to −2.6	3E-10 to 0.002	BT_RS01555 to 60	BT0317 to 18	TonB-dependent receptor, SusD/RagB family nutrient-binding outer membrane lipoprotein
−3.5 to −2.7	6E-05 to 0.004	BT_RS01700 to 05	BT0348 to 49	Alpha-*N*-arabinofuranosidase, glycoside hydrolase family 127 protein
−8.4 to −4.1	3E-09 to 2E-05	BT_RS01755 to 80	BT0360 to 65	Family 43 glycosylhydrolase, ragb/susd family nutrient uptake outer membrane proteins, tonb-dependent receptor, tonb-dependent receptor, hypothetical protein
−4.74	0.007	BT_RS01790	BT0367	Arabinan endo-1,5-alpha-L-arabinosidase
−2.8 to −2.6	6E-04 to 0.021	BT_RS02145 to 65	BT0438 to 40	Alpha-*N*-acetylglucosaminidase, tonb-dependent receptor, ragb/susd family nutrient uptake outer membr. Protein
−2.6	0.021	BT_RS02165	BT0442	DUF4855 domain-containing protein
−6.1 to −2.6	9E-04 to 0.025	BT_RS02190 to 205	BT0446 to 49	DUF5009 domain-containing protein, family 10 glycosylhydrolase, calcineurin-like phosphoesterase C-terminal domain-containing protein, family 10 glycosylhydrolase
−7.1 to −2.5	8E-04 to 0 0.154	BT_RS02215 to 30	BT0451 to 54	RagB/SusD family nutrient uptake outer membrane protein, TonB-dependent receptor, sugar porter family MFS transporter
−4.22	0.353	BT_RS02670	BT0545	P-II family nitrogen regulator
−3.5 to −2.5	0.09 to 0.326	asnB, BT_RS02700 to 05	BT0551 to 52	Asparagine synthase B, glutamate synthase subunit beta
−2.96	0.014	BT_RS02775	BT0566, BT_0566	Hypothetical protein
−3.4 to −2.6	1E-05 to 0.007	rsxC, BT_RS03050	BT0618 to 22	Electron transport complex subunit rsxc, rnfabcdge type electron transport complex sub D, rnfabcdge type electron transport complex sub G, electron transport complex subunit E, electron transport complex subunit rsxa
−9.3	0.132	BT_RS03280	BT0662	*O*-Antigen ligase family protein
−4.2	0.353	BT_RS03290	BT0664	6-Bladed beta-propeller
−6.8 to −3.1	0.002 to 0.015	BT_RS03390 to 400	BT0678 to 80	TolC family protein, efflux RND transporter periplasmic adaptor subunit, CusA/CzcA family heavy metal efflux RND transporter
−3.6 to −2.5	6E-07 to 2E-04	BT_RS03430, 35 (hcp)	BT0686 to 87	Uracil-xanthine permease, hydroxylamine reductase
−4.2	0.353	BT_RS03535	BT0706	*N*-acetylmuramoyl-l-alanine amidase
−5.9 to −2.9	6E-04 to 0.241	BT_RS03550 to 55	BT0709 to 10	DUF3987 domain-containing protein, hypothetical protein
−3.6	0.02	BT_RS03775	BT0755	RagB/SusD family nutrient uptake outer membr. protein
−4.2	0.117	arsAB, BT_RS04010 to 15	BT0802 to 03	Arsenical pump-driving ATPase, ACR3 family arsenite efflux transporter
−3.0	0.08	BT_RS04210 to 15	BT0841 to 42	Hypothetical protein
−5.9	0.241	BT_RS04295	BT0857	TolC family protein
−2.5	0.135	BT_RS04315	BT0861	ABC transporter permease
−3.0	5E-06	BT_RS04345	BT0867	TonB-dependent receptor
−2.8	3E-05	BT_RS04355	BT0869	Lipocalin family protein
−2.9	0.05	BT_RS04620 to 25	BT0918 to 19	IS*3* family transposase, hypothetical protein
−16.1 to −2.5	0.065 to 0.57	BT_RS04690 to 700	BT0933 to 35	Hypothetical proteins
−2.5	0.57	BT_RS04705	BT0937	Hypothetical protein
−3.1	0.248	BT_RS04730	BT0940	Hypothetical protein
−2.6	0.018	BT_RS04760	BT0945	Hypothetical protein
−4.2 to −2.5	0.353 to 0.57	BT_RS04770 to 75	BT0947 to 48	Tyrosine-type recombinase/integrase, DUF3871 family protein
−2.5	0.57	BT_RS04870	BT0967	DUF3098 domain-containing protein
−2.5	0.57	BT_RS04920	BT0977	Hypothetical protein
−7.6	0.175	BT_RS04960	BT0985	9-*O*-acetylesterase
−4.5	0.02	mgtA, BT_RS04975	BT0988	Magnesium-translocating P-type ATPase
−4.2	0.057	BT_RS05040	BT1001	Family 78 glycoside hydrolase catalytic domain
−2.8 to −2.5	0.113 to 0.57	BT_RS05160 to 65	BT1025 to 26	TonB-dependent receptor, DUF4957 domain-containing protein
−2.6	1E-04 to 4E-04	BT_RS05260 to 65	BT1046 to 47	SusC/RagA family TonB-linked outer membrane protein, SusD/RagB family nutrient-binding outer membr. lipoprotein
−5.4	0.072	BT_RS05280	BT1050	Discoidin domain-containing protein
−3.1	0.233	BT_RS05360	BT1067	Hypothetical protein
−2.7	0.008	BT_RS05390	BT1072	DUF4251 domain-containing protein
−7.6	0.175	BT_RS05720	BT1133	Sigma-70 family RNA polymerase sigma factor
−4.0	0.003	BT_RS05775	BT1143	YecH family protein
−4.2	0.353	BT_RS05900	BT1168	*N*-acetylmuramoyl-l-alanine amidase
−2.5	0.26	BT_RS06405	BT1269	Efflux RND transporter periplasmic adaptor subunit
−5.0 to −3.1	4E-05 to 0.001	BT_RS06435 to 45	BT1275 to 77	Rhamnulokinase, L-rhamnose mutarotase, L-fucose:H+ symporter permease
−7.8 to −3.3	3E-10 to 0. 021	BT_RS06460 to 85	BT1280 to 85	TonB-dependent receptor, SusD/RagB family nutrient-binding outer membr. lipoprotein, hypothetical protein, DUF1735 domain-containing proteins, endo-beta-N-acetylglucosaminidase
-4.0	0.005	BT_RS07015	BT1384	Pyridoxamine 5′-phosphate oxidase family protein
-2.5	0.57	BT_RS07035	BT1388	Multidrug efflux SMR transporter, cytochrome c biogenesis protein ccsa, cytochrome c biogenesis protein resb
−36.4 to −30.4	0	BT_RS07165 to 85	BT1414 to 18	Alginate export family protein, cytochrome c biogenesis protein ccsa, cytochrome c biogenesis protein resb, ammonia-forming cytochrome c nitrite reductase, cytochrome c nitrite reductase small subunit
−6.3 to −5.2	2E-13 to 7E-06	BT_RS07335 to 340	BT1448 to 49	Biotin/lipoyl-binding protein, acetyl-coa carboxylase biotin carboxylase subunit
−5.1	2E-12	BT_RS07345	BT1450	Acyl-coa carboxylase subunit beta
−4.2	0.353	BT_RS07405	BT1462	Hypothetical protein
−2.5	0.57	BT_RS07435	BT1468	TolC family protein
−3.1	0.222 to 0	BT_RS08345 to 50	BT1646 to 47	Glycosyltransferase family 2 protein, hypothetical protein
−4.2 to −2.5	0.57	BT_RS08660 to 70	BT1708 to 10	Sugar transferase, glycosyltransferase, EpsG family protein
−2.5	0.57	BT_RS08700 to 05	BT1716 to 17	CDP-glycerol glycerophosphotransferase family protein, lipopolysaccharide biosynthesis protein
−3.0	5E-06	BT_RS09005	BT1777	Hlycoside hydrolase family 95 protein
−4.8	0.095	BT_RS09070	BT1790	Hypothetical protein
−3.6 to −3.0	2E-09 to 3E-09	BT_RS09295 to 300 (hydEFG)	BT1834 to 37	4Fe-4S dicluster domain protein, [FeFe] hydrogenase H-cluster radical SAM maturase HydE, {FeFe] hydrogenase H-cluster maturation GTPase HydF
−7.6	0.175	BT_RS09480	BT1868	Tetratricopeptide repeat protein
−3.0	2E-07	BT_RS09725	BT1918	Arylsulfatase
−4.2	0.353	BT_RS09870	BT1947	DUF3408 domain-containing protein
−4.2	0.353	BT_RS10275	BT2029	Hypothetical protein
−2.6	0.002	BT_RS10620	BT2097	Glycoside hydrolase family 127 protein
−2.8	0.013	BT_RS10660	BT2105	GH92 family glycosyl hydrolase
−9.3	0.138	BT_RS10670	BT2107	TonB-dependent receptor
−2.9 to −2.6	2E-06 to 1E-04	BT_RS10915 to 25	BT2156 to 58	Sugar phosphate isomerase/epimerase, DUF1080 domain-containing protein, Gfo/Idh/MocA family oxidoreductase
−5.2	0.1	BT_RS11095	BT2193	Hypothetical protein
−3.7	3E-04	BT_RS11125	BT2199	GH92 family glycosyl hydrolase
−3.0	0.075	BT_RS11135	BT2201	RagB/SusD family nutrient uptake outer membr. protein
−3.1	0.1	BT_RS11145	BT2203	LamG domain-containing protein
−2.6	0.155	BT_RS11365	BT2247	Hypothetical protein
−7.3 to −3.0	2E-09 to 2E-06	BT_RS11405 to 10	BT2255 to 26	Acyltransferase, fumarate hydratase
−2.5	0.57	BT_RS11555	BT2285	Hypothetical protein
−7.6	0.175	traN, BT_RS11575	BT2289	Conjugative transposon protein tran
−2.5	0.57	traJ, BT_RS11595 to 600	BT2293 to 94	Conjugative transposon protein traj, DUF4141 domain-containing protein
−2.5	0.327	traG, BT_RS11625	BT2298 to 99	TraG family conjugative transposon ATPase, DUF4133 domain-containing protein
−2.5	0.57	BT_RS11670	BT2307	RteC domain-containing protein
−4.3	0.343	BT_RS11805	BT2335	Site-specific integrase
−2.7	0.23	BT_RS11825	BT2339	Hypothetical protein
−13.7 to −7.8	4E-14 to 3E-06	BT_RS12090 to 105	BT2392 to 95	IPT/TIG domain-containing protein, TonB-dependent receptor, RagB/SusD family nutrient uptake outer memb. protein, DUF4361 domain-containing protein
−2.8	0.046	BT_RS12130	BT2400	DNA-3-methyladenine glycosylase I
−4.8	4E-05	BT_RS12205	BT2414	4Fe-4S binding protein (FdxA)
−7.6	0.175	BT_RS12375	BT2442	OmpA family protein
−2.5	0.57	BT_RS12505	BT2469	Tyrosine-type recombinase/integrase
−2.5	0.056	BT_RS12805	BT2532	RagB/SusD family nutrient uptake outer memb. protein
−5.4	3E-04	BT_RS12945	BT2560	TonB-dependent receptor
−2.5	0.57	BT_RS13100	BT2589	SAM-dependent DNA methyltransferase
−2.5	0.57	BT_RS13105	BT2590	Hypothetical protein
−7.6	0.175	BT_RS13120 to 25	BT2593 to 94	DUF3872 domain-containing protein, conjugal transfer protein TraO
−5.9 to −2.6	0.346	BT_RS13120 to 25, traN	BT2593 to 95	DUF3872 domain-containing protein, conjugal transfer protein TraO, conjugative transposon protein TraN
−2.5		BT_RS13155	BT2600	
−7.6 to −4.2	0.178 to 0.353	BT_RS13165 to 75, traG	BT2602 to 03	Maturase, TraG family conjugative transposon ATPase, DUF4133 domain-containing protein
−31.2 to −3.1	3E-13 to 0.18	BT_RS13225 to 3310, ltrA	BT2614 to 32	YWFCY domain-containing protein, group II intron reverse transcriptase/maturase, sigma-54-dependent Fis family transcriptional regulator, response regulator, glycoside hydrolase family 97 protein, hypothetical protein, alpha-glucuronidase, glycoside hydrolase family 88 proteins, DUF4361 domain-containing protein, RagB/SusD family nutrient uptake outer memb. protein, SusC/RagA family TonB-linked outer memb. protein, IPT/TIG domain-containing protein, response regulator, GH92 family glycosyl hydrolase, endonuclease/exonuclease /phosphatase family protein, glycoside hydrolase family 125 protein
−2.5	0.57	BT_RS13340	BT2643	DUF4099 domain-containing protein
−4.2	0.352	BT_RS13375 to 80	BT2648 to 49	Prokaryotic E2 ligase family D protein, PRTRC system ThiF family protein
−4.2	0.353	BT_RS13395	BT2652	Helix-turn-helix domain-containing protein
−3.1 to −2.7	3E-04 to 0.003	BT_RS13455 to 75	BT2664 to 68	Substrate-binding domain-containing protein, biopolymer transporters ExbD, MotA/TolQ/ExbB proton channel family protein
−6.4 to −4.5	0 to 4E-08	rbsK, BT_RS14220	BT2804, BT_2804	Ribokinase, TonB-dependent receptor, RagB/SusD family nutrient uptake outer memb. protein, DUF4969 domain-containing protein, nucleoside hydrolase, multidrug DMT transporter permease
−4.2	0.352	BT_RS14270	BT2814	Dihydrodipicolinate synthase family protein
−11	0.107	BT_RS14485	BT2856	DUF5013 domain-containing protein
−4.2	0.353	BT_RS14490	BT2857	DUF4959 domain-containing protein
−3.5	0.065	BT_RS14495	BT2858	RagB/SusD family nutrient uptake outer memb. protein
−3.1	0.232	BT_RS14600 to 05	BT2879 to 80	CDP-glycerol glycerophosphotransferase family protein, NAD-dependent epimerase/dehydratase familyprotein
−3.1	0.222	BT_RS14615	BT2882	Glycosyltransferase
−4.3 to −2.5	0.343 to 0.347	BT_RS14625 to 30	BT2884 to 85	WbqC family protein, DegT/DnrJ/EryC1/StrS family aminotransferase
−2.5	0.57	BT_RS14650	BT2889	Helix-turn-helix transcriptional regulator
−4.2	0.353	BT_RS14675	BT2895	Family 43 glycosylhydrolase
−4.2	0.352	BT_RS14700	BT2900	Family 43 glycosylhydrolase
−14.2 to −3.1	0.002 to 0.241	BT_RS14710 to 30	BT2902 to 06	Hypothetical protein, DUF4959 domain-containing protein, TonB-dependent receptor, glycoside hydrolase family 99-like domain protein
−4.2 to −3.5	0.073 to 0.154	BT_RS14800 to 810	BT2920 to 22	TonB-dependent receptor, glycoside hydrolase family 88 protein, beta-galactosidase
−14.3 to −9.8	0.01 to 0.07	BT_RS15040 to 45	BT2968 to 69	TonB-dependent receptor, beta-galactosidase
−2.8	0.043	BT_RS15115	BT2982	Hypothetical protein
−2.5	0.57	BT_RS15130 to 140	BT2985 to 86	JAB domain-containing protein, tyrosine-type recombinase/integrase, hypothetical protein
−2.5	0.326	BT_RS15165	BT2991	Hypothetical protein
−4.2	0.353	BT_RS15195	BT2995	Relaxase/mobilization nuclease domain-containing protein
−8.3 to −2.6	1E-04 to 0.179	BT_RS15285 to 305	BT3012 to 16	TonB-dependent receptor, RagB/SusD family nutrient uptake outer memb. protein, DUF4959 domain-containing protein, discoidin domain-containing protein, TonB-dependent receptor
−9.5 to −3.0	5E-09 to 0.011	BT_RS15340 to 60	BT3024 to 28	TonB-dependent receptor, RagB/SusD family nutrient uptake outer memb. protein, glycosyl hydrolase, hypothetical protein, glycoside hydrolase family 130 protein
−4.9 to −4.0	9E to 080.015	BT_RS15440 to 50	BT3045 to 47	RagB/SusD family nutrient uptake outer memb. protein, TonB-dependent receptor IPT/TIG domain-containing protein
−2.5	3E-04	BT_RS15495	BT3055	Succinate dehydrogenase/fumarate reductase iron-sulfur subunit
−4.8	0.087	BT_RS15525	BT3061	DUF3575 domain-containing protein
−7.9 to −3.1	0,001 to 0.024	BT_RS15645 to 60	BT3087 to 90	Glycoside hydrolase family 66 protein, SusF/SusE family outer membrane protein, RagB/SusD family nutrient uptake outer memb. protein, TonB-dependent receptor
−3.8	0.009	BT_RS15675 to 80	BT3093 to 94	Arylsulfatase, family 43 glycosylhydrolase
−5.9 to −4.8	9E-07 to 0.241	BT_RS15725 to 35	BT3103 to 05	TonB-dependent receptor, hypothetical protein, BACON domain-containing protein
−2.6 to −2.5	0.372 to 057	BT_RS15880 to 90	BT3135 to 37	Site-specific integrase, site-specific integrase, hypothetical protein
−7.1	0.035	BT_RS15985	BT3157	RagB/SusD family nutrient uptake outer memb. protein
−3.7	0.024	BT_RS16035	BT3167	Helix-hairpin-helix domain-containing protein
−2.5	0.24	BT_RS16070	BT3174	TonB-dependent receptor
−3.0	3E-06	BT_RS16110	BT3182	Rubrerythrin family protein
−2.9	0.244	BT_RS16215 to 20	BT3202 to 03	RHS repeat-associated core domain-containing protein, hypothetical protein
−14.6 to −10.0	0.002 to 0.084	BT_RS16305 to 15	BT3221 to 23	DUF3244 domain-containing protein, DUF4848 domain-containing protein, hypothetical protein
−3.7	0.02	BT_RS16670	BT3291	Hypothetical protein
−3.2	0.028	BT_RS16695	BT3296	RagB/SusD family nutrient uptake outer memb. protein
−2.6	0.001	BT_RS16820	BT3322	Redoxin family protein
−2.9	0.005	BT_RS16855	BT3329	DUF4988 domain-containing protein
−11.1 to −5.3	0 to 4E-07	BT_RS16930 to 45	BT3344 to 47	DUF4973 domain-containing protein, RagB/SusD family nutrient uptake outer memb. protein, TonB-dependent receptor, IPT/TIG domain-containing protein
−19.4	0.044	BT_RS17260 to 265	BT3410	Heavy metal-binding domain-containing protein, hypothetical protein
−3.1	3E-06	BT_RS17300	BT3418	Thiol:disulfide interchange protein
−2.7	0.139	BT_RS17405	BT3439	Hypothetical protein
−3.0 to −2.7	0.004 to 0.065	BT_RS17570 to 80	BT3474 to 76	RagB/SusD family nutrient uptake outer memb. protein, TonB-dependent receptor, IPT/TIG domain-containing protein
−2.53	0.57	BT_RS17605	BT3481	DUF1793 domain-containing protein
−19.4	0.045	BT_RS17625	BT3485	DUF4973 domain-containing protein
−7.6	0.175	BT_RS17685 to 90	BT3498 to 99	DUF4956 domain-containing protein, DUF2490 domain-containing protein
−5.3	0.002	BT_RS17715	BT3505	TonB-dependent receptor
−4.2	0.353	BT_RS17740	BT3510	Hypothetical protein
−3.2	0.001	BT_RS18035	BT3572	Hypothetical protein
−2.7	0.086	BT_RS24405	BT3582	Hypothetical protein
−11.0	0.104	BT_RS18130	BT3593	SGNH/GDSL hydrolase family protein
−3.7	0.01	BT_RS18170	BT3601	SIS domain-containing protein
−4.2	0.353	BT_RS18420	BT3651	DUF3137 domain-containing protein
−3.2	5E-04	BT_RS18450	BT3657	Carbohydrate binding domain-containing protein
−3.1	0.222	BT_RS18475, BT_RS18480	BT3663	Hypothetical protein, glycoside hydrolase 43 family protein
−2.97	0.019	BT_RS18560	BT3680, BT_3680	TonB-dependent receptor
−4.2 to −2.9	1E-09 to 5E-05	BT_RS18645 to 70, susDEF	BT3698 to 703	Alpha-amylase SusG, DUF5115 domain-containing protein, SusF/SusE family outer membrane protein, starch-binding outer membrane lipoprotein SusD, TonB-dependent receptor, glycoside hydrolase family 97 protein
−2.53	0.57	BT_RS18900	BT3747, BT_3747	Hypothetical protein
−3.14	6E-05	BT_RS18915	BT3750, BT_3750	SusC/RagA family TonB-linked outer memb. protein
−6.4 to −3.4	9E-06 to 4E-05	BT_RS18920 to 30	BT3752 to 54	SusD/RagB family nutrient-binding outer memb. lipoprotein, endo-beta-*N*-acetylglucosaminidase, DUF1735 domain-containing protein
−4.6 to −2.8	6E-08 to 0.007	BT_RS19040 to 55	BT3775 to 78	Hypothetical proteins
−12.6 to −3.4	2E-08 to 9E-08	BT_RS19065 to 75	BT3780 to 82	Hypothetical protein, glycoside hydrolase family 125 protein, glycoside hydrolase family 88 protein
−22.3 to −2.9	0 to 2E-08	BT_RS19085 to 120	BT3784 to 92	GH92 family glycosyl hydrolase, hybrid sensor histidine kinase/response regulator transcription factor, IPT/TIG domain-containing protein, TonB-dependent receptor, RagB/SusD family nutrient uptake outer membrane protein, DUF4361 domain-containing protein, LamG domain-containing protei, glycoside hydrolase family 76 protein,
−5.3 to −3.5	2E-06 to 0.013	BT_RS19140 to 55	BT3796 to 99	Sulfatases, alpha-galactosidase, alpha-L-fucosidase
−2.7	0.007	BT_RS19275	BT3823	Ferritin
−6.4 to −2.8	4E-11 to 2E-05	BT_RS19460 to 80	BT3854 to 58	SusC/RagA family TonB-linked outer membrane protein, RagB/SusD family nutrient uptake outer membrane protein, DUF3823 domain-containing protein, hypothetical protein, GH92 family glycosyl hydrolase
−2.7	5E-04	BT_RS19495	BT3861	DUF4972 domain-containing protein
−3.1 to −2.7	7E-06 to 2E-04	BT_RS19695 to 705	BT3902 to 04	ABC transporter permeases, HlyD family secretion protein
−2.7 to −2.6	0.038 to 0.106	BT_RS19750 to 55	BT3913 to 14	DUF5034 domain-containing protein, hypothetical protein
–3.0 to –2.8	5E-06 to 0.099	BT_RS19935 to 55	BT3951 to 53	Helix-turn-helix domain-containing protein, TonB-dependent receptor, RagB/SusD family nutrient uptake outer membrane protein
−4.8	0.037	BT_RS19955	BT3955	Hypothetical protein
–3.6 to –2.6	4E-10 to 7E-05	BT_RS19970 to 75	BT3958 to 59	TonB-dependent receptor, RagB/SusD family nutrient uptake outer membrane protein
−3.2	2E-07	BT_RS19985	BT3961	DUF4302 domain-containing protein
−2.7	0.003	BT_RS20110	BT3985	Hypothetical protein
−2.6	0.043	BT_RS20125	BT3988	DUF4989 domain-containing protein
−2.5	0.57	BT_RS20270	BT4016	Hypothetical protein
−4.2	0.353	BT_RS20305 to 310	BT4023 to 24	Tyrosine-type recombinase/integrase, hypothetical protein
−2.5	0.57	BT_RS20325 to 330	BT4027 to 28	Hypothetical protein
–5.3 to –3.3	8E-12 to 3E-04	BT_RS20380 to 90	BT4038 to 40	RagB/SusD family nutrient uptake outer membrane protein, TonB-dependent receptor, hypothetical proteins
–6.4 to –3.4	9E-06 to 0.011	T_RS20585 to 610	BT4078 to 83	Hypothetical proteins, IPT/TIG domain-containing protein, RagB/SusD family nutrient uptake outer membrane protein, DUF4361 domain-containing protein
−2.5	0.57	BT_RS20625	BT4086	DUF5004 domain-containing protein
–14.3 to –3.4	1E-04 to 0.353	BT_RS20740 to 65	BT4109 to 14	Pectinesterase, pectin esterase, DUF5123 domain-containing protein, TonB-dependent receptor
−7.5 to –3.5	0.007 to 0.105	BT_RS20950 to 55	BT4152 to 53	Beta-galactosidase, exopolygalacturonase
−6.3 to –3.3	4E-06 to 0.005	BT_RS21010 to 35	BT4164 to 69	SusC/RagA family TonB-linked outer membrane protein, DUF5108 domain-containing protein, hypothetical proteins, TonB-dependent receptor
−3.1 to –2.5	0.074 to 0.23	BT_RS21070 to 75, rhaM	BT4176 to 77	Glycoside hydrolase family 88 protein, L-rhamnose mutarotase
−3.2	0.047	BT_RS21090	BT4180	Sialate *O*-acetylesterase
−3.2	0.018	BT_RS21195 to 200, miaA	BT4202 to 03	Hypothetical protein, tRNA (adenosine(37)-N6)-dimethylallyltransferase MiaA
−2.5	0.221	BT_RS21545	BT4268	RagB/SusD family nutrient uptake outer membrane protein
–17.0 to–8.2	1E-14 to –0.103	BT_RS21680 to 705	BT4294 to 99	Hypothetical protein, discoidin domain-containing protein, DUF4361 domain-containing protein, RagB/SusD family nutrient uptake outer membrane protein, TonB-dependent receptor, hypothetical protein
−4.2	0.015	BT_RS21995	BT4358	RagB/SusD family nutrient uptake outer membrane protein
−2.5	0.57	BT_RS22120	BT4384	Winged helix-turn-helix domain-containing protein
−2.9	0.071	BT_RS22160	BT4392	IS*4*-like element ISBthe3 family transposase
−2.7	0.013	BT_RS22225 to 30	BT4406 to 07	Hypothetical protein, DUF1735 domain-containing protein
−2.5	0.57	BT_RS22300 to 05	BT4421 to 22	Hypothetical proteins
−2.5	0.57	BT_RS22385	BT4438	Hypothetical protein
−3.2 to –2.5	0.011 to 0.147	BT_RS22435 to 40	BT4448 to 49	Gfo/Idh/MocA family oxidoreductases
−3.1 to –2.8	0.001 to 0.007	BT_RS22495 to 505	BT4460 to 62	Carboxypeptidase-like regulatory domain-containing protein, sigma-70 family RNA polymerase sigma factor, 1-acyl-sn-glycerol-3-phosphate acyltransferase
−2.5	0.57	BT_RS22810 to 15	BT4523 to 24	Restriction endonuclease subunit S, helix-turn-helix domain-containing protein
−3.1	0.017	BT_RS22820	BT4526	Metallophosphoesterase
−3.0	0.028	BT_RS23090	BT4581, BT4581	Glycoside hydrolase family 97 protein
−4.2	0.352	BT_RS23275 to 80	BT4618 to 19	Helix-turn-helix domain-containing protein, VirE protein
−4.2	0.353	BT_RS23290 to 95	BT4621 to 22	MobC family plasmid mobilization relaxosome protein, relaxase/mobilization nuclease domain-containing protein
−3.8 to –2.9	7E-06 to 0.004	BT_RS23480 to 90	BT4660 to 62	TonB-dependent receptor, DUF4958 family protein, heparin-sulfate lyase
−6.6	0.005	BT_RS23640	BT4693	Efflux RND transporter periplasmic adaptor subunit
–8.4 to –4.0	0.001 to 0.002	BT_RS23645 to 50	BT4694 to 95	CusA/CzcA family heavy metal efflux RND transporter, TolC family protein
−4.8	0.098	BT_RS23720	BT4708	SusD/RagB family nutrient-binding outer memb. lipoprotein
−2.5	0.349	BT_RS23725	BT4709	Glycosyl hydrolase
–6.9 to –2.9	2E-05 to 0.152	BT_RS23800 to 805	BT4724 to 25	TonB-dependent receptor and RagB/SusD outer membrane protein
−2.6	0.053	BT_RS23815	BT4727	Glycerophosphodiester phosphodiesterase family protein
−2.5	0.57	BT_RS23915	BT4746	DNA-binding protein
−4.8	0.097	BT_RS23945	BT4752	DUF3945 domain-containing protein
−7.6	0.175	BT_RS24010	BT4764	Hypothetical protein
−4.2 to –2.5	0.326 to 0.353	BT_RS24065 to 75, traK	BT4776 to 78	Conjugative transposon protein TraK, hypothetical protein, conjugal transfer protein
−2.5	0.57	traN, BT_RS24085	BT4780	Conjugative transposon protein TraN

a The order of the listed genes is according to the old locus tag. Genes clustering together on the chromosome (potential operons) are shaded in gray. The strain is under NC_004663 (CDS).

b Ratio of gene expression in bacteria grown under iron-depleted compared with iron-rich conditions.

c *P* value from an experiment performed in four independent biological replicates.

Two genes, namely, BT0519 and BT0520, coding for hemerythrin domain-containing protein and a response regulator/transcription factor were upregulated by 16.3- and 20-fold, respectively. An operon coding for carbohydrate metabolism composed of BT1658 to BT1660 was upregulated by 15.4-, 16.8-, and 20-fold, respectively. Also, BT2818 to BT2825 coding for a locus containing the SusD/SusC system was upregulated 4.5- to 22.5-fold, depending on the gene.

Oxidative stress protection mechanisms, such as gene BT0028 coding for alkaline phosphatase, were upregulated 15.4-fold. BT2811 coding for alkyl hydroperoxide reductase subunit F was also upregulated by 8-fold. Finally, gene BT4815 coding for the DNA starvation/stationary phase protection protein (Dps) was upregulated by 5.8-fold.

Finally, the iron-independent metabolism operon BT0516 to BT0517 coding for a flavodoxin (FldA; BT0517) and DUF2023 protein (BT0516) were upregulated 326- and 598-fold, respectively. This operon was also reported to be highly upregulated in the colitis model (2) ((Table 7).

**TABLE 7:** Clinical correlation of iron-dependent regulation^*a*^

−Fe/+Fe^*b*^	Gene name^*c*^	Colitis study^*d*^	Protein annotation	Presence of the gene in^*e*^:
1.898914	BT_0159	2.111094	VIT1/CCC1 transporter family protein	Bt
8.06201	BT_0224	9.704741	Hypothetical protein	Bt
4.40866	BT_0225	11.11973	Hypothetical protein	Bt, Pi
4.883878	BT_0226	10.46389	Hypothetical protein	Bt
3.419034	BT_0227	7.563568	Porin family protein	Bt
1.633861	BT_0239	−1.05735	DUF4369 domain-containing protein	Bt, Pg, Pi
108.9247	BT_0491	18.86303	DUF2149 domain-containing protein, MotA/TolQ/ExbB proton channel family protein	Bt, Pg, Pi
186.9863	BT_0492	28.50811	DUF2149 domain-containing protein, MotA/TolQ/ExbB proton channel family protein	Bt, Pg, Pi
136.0809	BT_0493	16.425	Hypothetical protein	Bt, Pg, Pi
282.4144	BT_0496	24.69941	TonB-dependent receptor	Bt, Pg, Pi
290.8443	BT_0497	29.40818	HmuY family protein	Bt
263.7668	BT_0498	31.73038	Hypothetical protein	Bt, Pg,
2.392774	BT_0503	8.93965	Hypothetical protein	Bt, Pg
2.430991	BT_0504	4.850745	TonB-dependent receptor	Bt, Pg, Pi
51.24636	BT_0507	5.508571	TetR/AcrR family transcriptional regulator	Bt, Pi, Pi
675.5204	BT_0508	15.40959	TetR/AcrR family transcriptional regulator	Bt, Pi
516.2514	BT_0509	11.91826	ABC transporter ATP-binding protein	Bt,
37.04651	BT_0515	3.708254	Quinol oxidase	Bt, Pg, Pi
598.3778	BT_0516	27.22851	DUF2023 family protein	Bt, Pg, Pi
325.7	BT_0517	29.3	Flavodoxin	Bt, Pg, Pi
19.95236	BT_0519	9.597707	Hemerythrin domain-containing protein, response regulator	Bt, Pg, Pi
16.32074	BT_0520	9.573912	Hemerythrin domain-containing protein, response regulator transcription factor	Bt
2.014698	BT_0523	4.325417	Sugar transferase	Bt
2.142719	BT_0524	1.586668	Response regulator	Bt,
2.007205	BT_0532	7.851734	Anthranilate synthase component I family protein	Bt, Pg
−4.22	BT_0545	−174	P-II family nitrogen regulator	Bt
−2.5	BT0551	−167.1	Asparagine synthase	Bt
−3.5	BT0552	−1,267	Glutamate synthase beta subunit	Bt
−1.99	BT0553	−290	Glutamate synthase large subunit	Bt
3.711937	BT_0922	4.113622	PepSY-like domain-containing protein	Bt, Pg
5.042992	BT_0923	3.241869	PepSY-like domain-containing protein	Bt
2.027212	BT_0995	1.799071	PAS domain-containing sensor histidine kinase, helix-turn-helix domain-containing protein	Bt
3.760234	BT_1043	10.62892	SusD/RagB family nutrient-binding outer membrane protein	Bt
8.318422	BT_1045	10.67078	DUF1735 domain-containing protein	Bt, Pg, Pi
3.099403	BT_1138	−1.56722	hypothetical protein, site-specific integrase	Bt
2.279712	BT_1597	3.318077	Tetratricopeptide repeat protein, HAMP domain-containing histidine kinase	Bt
3.294873	BT_1895	35.68394	Hypothetical protein	Bt
3.128725	BT_1896	40.56019	Leucine-rich repeat protein	Bt, Pg
3.320197	BT_2038	5.169461	Efflux RND transporter periplasmic adaptor subunit	Bt, Pg
2.477183	BT_2039	6.533055	CusA/CzcA family heavy metal efflux RND transporter	Bt, Pg
2.549452	BT_2040	4.430047	TolC family protein	Bt
489.3635	BT_2063	34.05744	PepSY domain-containing protein	Bt
676.0915	BT_2064	34.84073	DUF4374 domain-containing protein	Bt, Pi
662.945	BT_2065	16.46505	TonB-dependent receptor	Bt, Pi
3.463556	BT_2450	26.23424	Hypothetical protein	Bt
62.68564	BT_2473	14.21087	Fimbrillin family protein	Bt
103.9787	BT_2475	29.12251	Cytochrome c biogenesis protein ccsa, hypothetical protein	Bt
225.3287	BT_2476	28.66125	Hypothetical protein	Bt
146.1537	BT_2477	25.62196	Porin	Bt
133.2681	BT_2478	30.08073	Thiol oxidoreductase	Bt
230.1729	BT_2479	32.79333	Peptidase M75	Bt
283.9951	BT_2480	26.77281	Hypothetical protein	Bt
323.7068	BT_2482	20.39152	Helix-turn-helix transcriptional regulator	Bt
2.264999	BT_2778	1.409648	Sigma-70 family RNA polymerase sigma factor	Bt
−2.26543	BT_3053	−3.48253	Succinate dehydrogenase/fumarate reductase cytochrome b subunit	Bt, Pg, Pi
−2.29584	BT_3054	−2.86087	Fumarate reductase/succinate dehydrogenase flavoprotein subunit	Bt, Pg, Pi(p)
65.02367	BT_3625	15.21476	DUF4857 domain-containing protein	Bt
36.69843	BT_3626	14.07917	hypothetical protein	Bt
39.85516	BT_3627	18.25618	ABC transporter ATP-binding protein	Bt,
38.27088	BT_3628	17.59845	PepSY-associated TM helix domain-containing protein	Bt
43.33606	BT_3629	17.27476	PepSY-associated TM helix domain-containing protein	Bt
97.71608	BT_3630	19.14698	Fimbrillin family protein	Bt
128.0625	BT_3631	14.74463	Hypothetical protein, DUF4876 domain-containing protein	Bt
125.3197	BT_3632	16.11913	Hypothetical protein, DUF4876 domain-containing protein	Bt
112.1206	BT_3633	18.93561	TonB-dependent receptor	Bt
5.035874	BT_3969	3.185176	CusA/CzcA family heavy metal efflux RND transporter	Bt
2.017194	BT_4227	13.80192	Mfa1 fimbrilin C-terminal domain-containing protein	Bt,
2.305598	BT_4233	9.275554	DUF3845 domain-containing protein	Bt
3.306148	BT_4234	9.429591	DUF3575 domain-containing protein	Bt, Pi
2.182647	BT_4236	−1.25097	Response regulator	Bt
4.047878	BT_4571	3.342238	Hypothetical protein, RNA polymerase sigma factor	Bt
5.844437	BT_4715	18.7437	DNA starvation/stationary phase protection protein	Bt, Pg, Pi

a Light and dark gray shaded blocks of genes convey clustering on a genome of B. thetaiotaomicron VPI BT5482.

b Fold change in this study (−Fe/+Fe).

c Name of B. thetaiotaomicron VPI BT5482 genes.

d Fold change in colitis study (2).

e Bt, B. thetaiotaomicron, Pg, P. gingivalis, Pi, P. intermedia.

A total of 444 genes were downregulated 2.5-fold. The regulated genes were arranged into 51 operons. Among the most drastically downregulated operons was the operon BT1414 to BT1418 that codes for dissimilative nitrite reduction to ammonia and includes the *nrfAH* (BT1417 to BT1418) and cytochrome reductase preceded by the cytochrome c biogenesis coding genes *ccsA* (BT1415) and *ccsB* (BT1416) (Table 6, Fig. 3I). That operon also included a gene coding for an alginate export family protein (BT1414). It is predicted to be regulated by a Crp-like regulator encoded by BT1413.

To extend the nitrogen regulation cycle, a gene coding for nitrogen-fixing protein NifU (BT0174) and *hcp* (BT0687) coding for putative hydroxylamine reductase (Hcp) (downregulated [−3.6-fold]) were also downregulated. The gene adjacent to *hcp* coding for uracil-xanthine permease (BT0686) was also downregulated by 2.5-fold, suggesting the two genes form an operon. *hcp* is predicted to be regulated by HcpR which is encoded by BT0688.

Genes encoding iron-dependent metabolic mechanisms, including the succinate dehydrogenase/fumarate reductase (BT3055) and RnfABCDGE electron transport complex (BT0618 to BT0622), were downregulated by 2.5- to 3.4-fold, depending on the gene. A gene encoding asparagine biosynthesis (BT0551) was downregulated by 2.5-fold and a locus coding for glutamate synthase (BT0552 to BT0553) was downregulated by 3.5-fold (Fig. 3L). Finally, locus *hydGEF* (BT1834 to BT1837) coding for a 4Fe-4S cluster containing hydrogenase was downregulated by 2.9-to 3.6-fold, depending on the gene.

Iron homeostasis- and oxidative stress-encoding genes, including a ferritin coding gene (*ftn*; BT3823) and a rubrerythrin family protein coding gene (BT3182), were downregulated by 2.7- to 3-fold, depending on the gene (Table 6). Of interest was also locus-encoding sulfatases, BT3796 to BT3799, downregulated by 3.5- to 5.3-fold depending on the gene (Table 6).

A large number of operons coding for two-component Sus-like transporters systems that contained the gene coding for TonB-dependent receptor as well as the Rag/Sus family nutrient uptake protein (BT4724 to BT4725, BT4294 to BT4299, BT4038 to BT4040, BT3958 to BT3959, BT3951 to BT3953, BT3854 to BT3858, BT3786 to BT3792, BT3344 to BT3347, BT3087 to BT3090, BT3045 to BT3047, BT3024 to BT3028, BT3012 to BT3016, BT2804 to BT2809, BT2614 to BT2632, BT2392 to BT2395, BT1280 to BT1285, BT1046 to BT1047, BT0438 to BT0440, BT0360 to BT0365, and BT0317 to BT0318) were downregulated (Table 6). Finally, conjugative transposon coding genes *traN*, *traM*, and *traK* (BT0086 to BT0089) were also downregulated by 2.5- to 7.6-fold, depending on the gene.

Overall, iron/hemin uptake, carbohydrate metabolism, oxidative stress, and iron-independent metabolism were upregulated. Downregulated were genes that code for proteins containing iron cofactors, such as metabolic enzymes (*nrfAH*, *ccsA*, *hcp*, *rnf*, *and hyd*) and oxidative stress (*rbr* and *ftn*), as well as TonB and RagA/Sus transport systems. Lower iron levels also reduced the expression of genes involved in DNA mobilization.

### Iron-dependent stimulon of *Bacteroidetes*.

**FIG 4:**
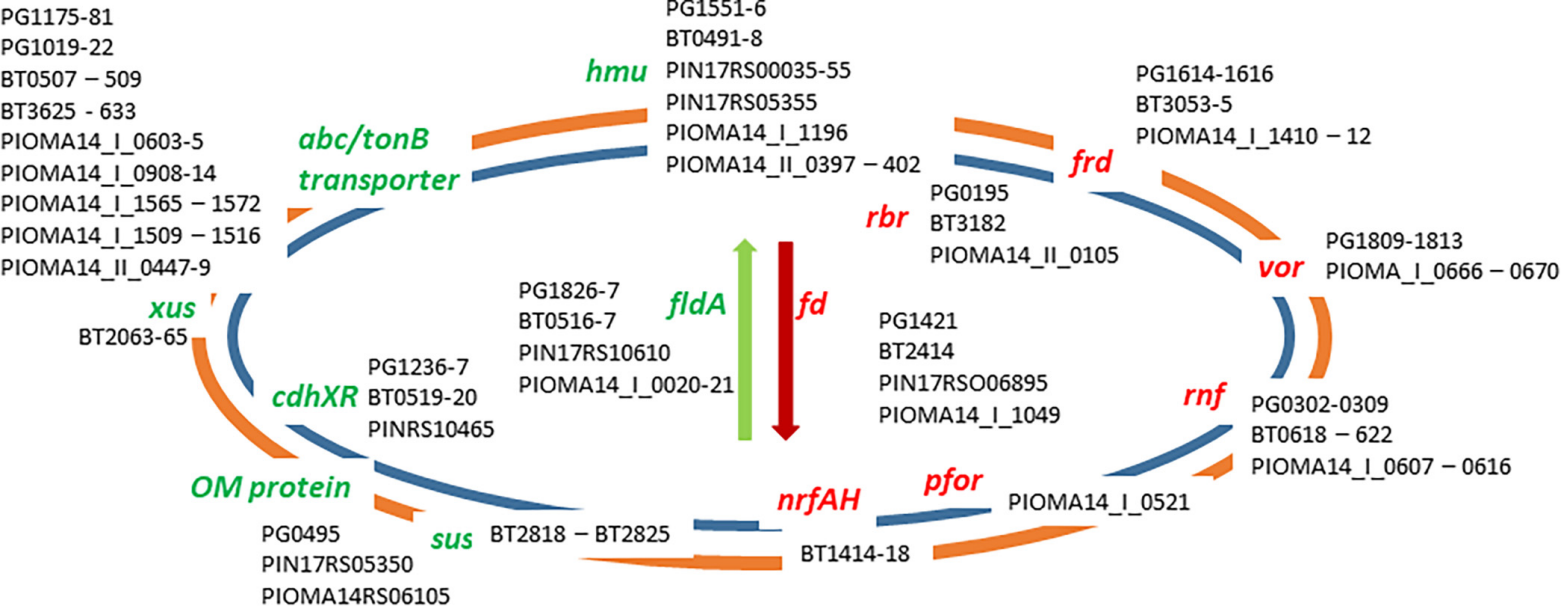
Schematic representation of iron-dependent gene regulation in *Bacteroidetes*. In bacteria grown under iron-limited conditions genes, that are upregulated are shown in green, while the downregulated ones are shown in red.

A comparison of the regulated genes across the three bacterial species analyzed in our study has identified many common genes (Fig. 4) (similarity in gene sequence and predicted functions) (Fig. 1 to 3). Furthermore, many of the genes had a similar genomic organization in the three bacterial species studied. Among the commonly regulated operons was the *hmu* gene encoding a hemin update mechanism (Table 1, 3, and 5, Fig. 1A). Also, the flavodoxin-encoding gene was highly upregulated in all three bacterial species tested (Fig. 1B). The locus is composed of two genes, as follows: *fldA* and a downstream gene coding for a hypothetical protein. In *B. thataiotaomicron*, the *fldA* gene and the downstream gene (BT0517 and BT0516) are predicted to form an operon. Such a prediction is further substantiated by the significant upregulation of both genes (Table 5). Since both genes are also present in the oral bacteria, it is likely that they are also cotranscribed. Two operons containing TonB-dependent receptors were upregulated in three (Fig. 1C) and two (Fig. 1D) bacteria, respectively. Drastically upregulated in oral species (P. gingivalis and P. intermedia) was PG0495 (upregulated 87-fold) and PIOMA14_RS06105 (ChI; upregulated 104-fold) (in P. intermedia 17, PIN17_RS05350) (Fig. 1E, Fig. S1). This gene encodes a T9SS C-terminal target domain protein. Of note, in P. intermedia, this locus is located next to *hmuY* (Fig. 1E).

There were also several commonly downregulated genes. The iron-based metabolism-coding genes, including the operon *sdhABC* coding for succinate dehydrogenase/fumarate reductase (Fig. 2A), the *rnf* gene coding for the electron transport chain (Fig. 2B), and *vor* gene coding for 2-oxoglutarate ferredoxin oxidoreductase (Fig. 2C), were downregulated in all three bacterial species tested. The rubrerythrin-encoding gene *rbr* was more drastically downregulated in P. gingivalis and P. intermedia (PG0195 and PIOMA14_II_0105 with 46- and 22.3-fold change, respectively) than in B. thetaiotaomicron (BT3812 downregulated by 3-fold) (Fig. 2D, Table 2, 4, and 6). It is noteworthy that B. thetaiotaomicron encodes another rubrerythrin (BT0216) that is proceeded by BT0215 coding for a putative iron uptake regulatory protein, Fur, but that locus was not significantly affected by iron. Also, the *ahp* locus coding for the alkylhydroperoxide reductase (Fig. 3B) was found to be upregulated in P. gingivalis and B. thetaiotaomicron.

Among other significantly regulated genes was the BT2063 to BT2065 locus encoding the xenosiderophore acquisition mechanism (2). That locus is present only in *B. thataiotaomicron* and is not found in P. gingivalis and P. intermedia 17 (Fig. 3A). Also, present only in B. thetaiotaomicron was the locus BT0552 to BT0553 coding for glutamate synthase as well as the locus BT1834 to BT1837 encoding the hydrogenase system. Another locus of interest, the methyltransferase-encoding locus, was upregulated (Fig. 3C) but only in P. gingivalis W83. Finally, the *nrfAH* locus encoding nitrite reductase was found in all three bacteria tested (Fig. 3D). However, it was found to be drastically affected by iron depletion only in B. thetaiotaomicron (Table 6). Of interest was the family of DUF1661 domain protein-encoding genes that were present only in P. gingivalis (see Fig. S4 in the supplemental material). This group is a family of genes coding for small proteins that may have unique functions in the adaptation of P. gingivalis to iron depletion.

Overall, very striking was the drastic upregulation of flavodoxin-encoding genes with a concomitant reduction of ferredoxin-encoding genes. Consistently, many iron-based metabolic proteins were downregulated. Iron uptake mechanisms were upregulated, possibly to alleviate the iron deficit in the cell (Fig. 4).

### Nitrite reduction in B. thetaiotaomicron decreases under iron-limiting conditions.

**FIG 5:**
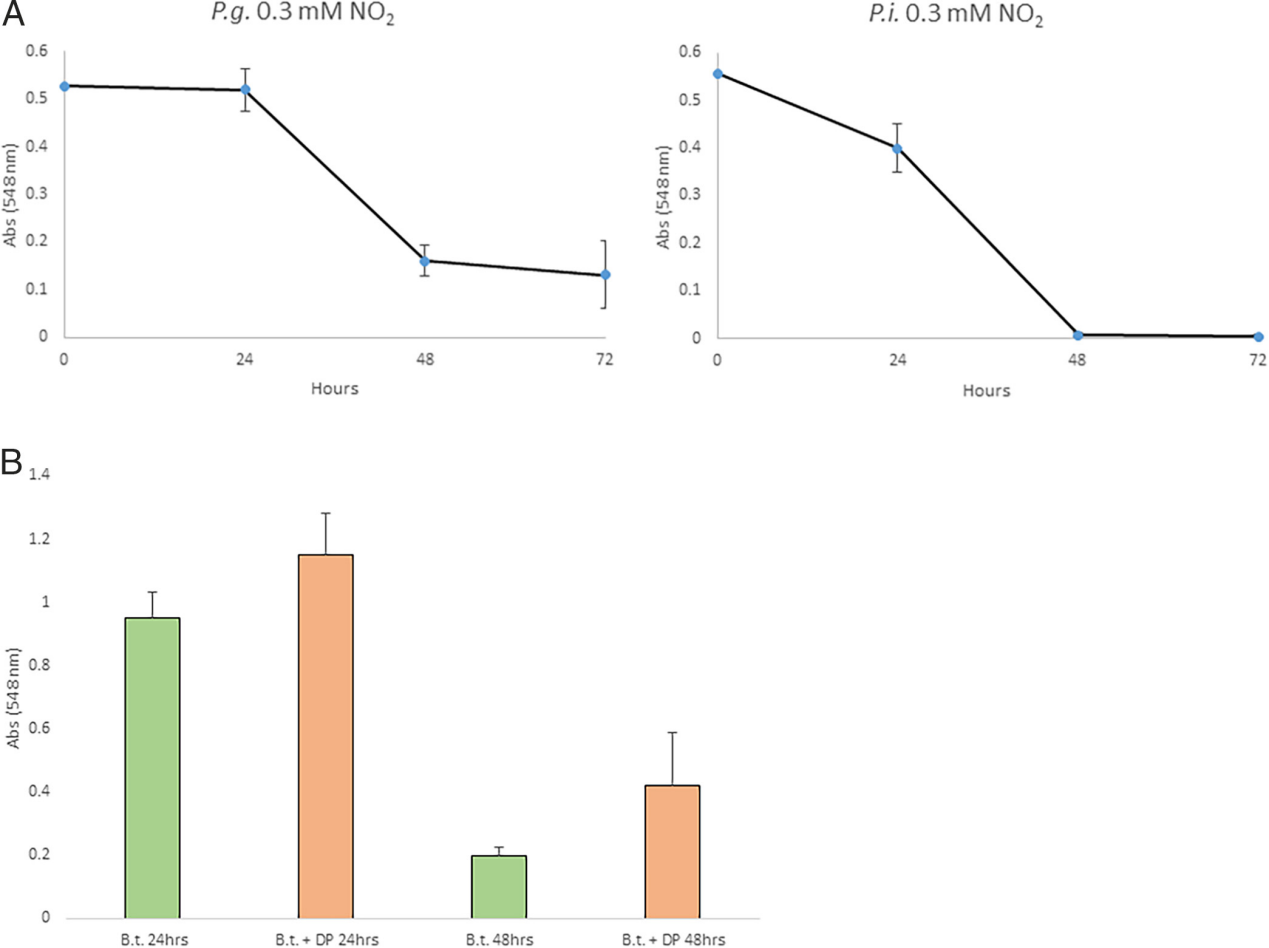
Bacteroides have the ability to reduce nitrite. (A) B. thetaiotaomicron *VPI* VPI BT5482 Δ*tdk* was grown in the presence and absence of 2 mM sodium nitrite in TSB medium with and without 150 μM DP. (B) P. gingivalis W83 and P. intermedia OMA14 were grown in the presence and absence of 0.3 mM sodium nitrite in BHI medium. Growth was monitored by measuring OD_660_, and levels of nitrite were determined with the Griess reagent.

While all the investigated bacterial species carry genes coding for nitrite reduction, the ammonia-forming cytochrome c nitrite reductase system NrfAH (Fig. 3D), the ability of the bacteria to utilize nitrite has not been demonstrated. Here, we show that all three bacterial species reduce nitrite levels present in the culture mixture (Fig. 5A and B). This ability differs among the species where B. thetaiotaomicron is the most efficient one in nitrite reduction. It is able to reduce 2 mM nitrite present in culture medium to undetectable levels within 48 h (Fig. 5B). P. intermedia OMA14 ranked 2nd in its ability to reduce nitrite, while P. gingivalis W83 was observed to complete the reduction of 0.3 mM nitrite to undetectable levels within 72 h (Fig. 5A). While iron depletion decreased the ability of B. thetaiotaomicron to reduce nitrite (*P* = 0.065 at a 48-h time point) (Fig. 5B), it had no effect on nitrite reduction by P. gingivalis and P. intermedia (results not shown). These findings further functionally verify the data obtained through our transcriptome sequencing analysis where a significant reduction in B. thetaiotaomicron transcript levels is observed under iron-limiting conditions.

### Clinical correlation of iron-dependent regulation in *Bacteroidetes*.

There was a significant overlap between genes upregulated in B. thetaiotaomicron during colitis (2) and genes upregulated under iron-deficient conditions in our study (Table 7). Sixty-eight genes where many were organized into operon structures (15 operon-like organizations) were identified to overlap between the two studies (Table 7). Several loci were present in all three bacteria tested.

First, the locus coding for a flavodoxin (FldA; BT0517) and DUF2023 protein (BT0516) was drastically upregulated in both our model and the colitis model (Table 5 and 7) (2). The P. gingivalis flavodoxin encoding gene *fldA* was found to be one of the most highly expressed genes in microbiomes derived from human subjects with periodontitis indicating that in the extracellular inflamed environment genes upregulated by iron deficiency are also highly expressed (19).

An additional significantly upregulated locus both in our study and during colitis is the *hmu* locus (Table 7). Furthermore, the BT0507 to BT0509 locus coding for the TetR/AcrR family transcriptional regulator and two ABC transporter ATP-binding proteins were also upregulated in our study as well as during colitis (Table 7, Fig. 1D). It is noteworthy that two copies of a similar operon were found on the genomes of P. intermedia (while the copy on the small chromosome was identical to that of the other bacteria studied), and the copy on the large chromosome included a distinct gene (the ABC-transporter coding gene) (Fig. 1D, ii). A similar locus is also present in P. gingivalis where the gene coding for the ABC transporter is followed by a gene coding for an outer membrane lipoprotein sorting protein (PG1179) and hypothetical protein (PG1178). An interesting finding was the PG1177 coding for a transposase followed by PG1176 to PG1175 encoding orthologs of BT0508 to BT0509 (Fig. 1D, ii). A mobile element-encoding gene, BT1138, that has similar genes in the other two bacteria tested was also regulated in both studies. In addition, the locus BT3053 to BT3055 encoding succinate dehydrogenase/fumarate reductase SdhABC was downregulated in both studies (Table 7, Fig. 2A). Finally, BT4715 *dps* coding for a DNA starvation/stationary phase protection protein is present in all three bacteria investigated in our study. It is of interest that in B. thetaiotaomicron upstream of *dps* is an *oxyR* (BT4716) facing in an opposite direction (Table 7), indicating that it may play a role in *dps* expression.

There were B. thetaiotaomicron loci that were regulated by iron levels and colitis and had counterparts in only one of the other bacteria investigated in our studies. The BT0503 to BT0504 locus has a similar composition in B. thetaiotaomicron and P. gingivalis where the first gene coding for the hypothetical protein (BT0503) is the same in both bacteria and is followed by a TonB-encoding gene (BT0504 and PG2008), which although they are different in sequence, both code for a functionally similarly TonB-dependent protein. Similarly, the BT0519 to BT0520 codes for a two-component hemerythrin-domain regulatory protein (Table 7, Fig. 3F). The operon is also present in P. gingivalis W83 and codes for the CdhR regulator (20). In addition, the BT0922 to BT0923 locus coding for a PepSY-like domain-containing protein as well as the BT2038 to BT2040 locus encoding a heavy metal efflux transporter system were present in both *B. thataiotaomicron* and P. gingivalis (Table 7).

Finally, there were loci regulated by iron and colitis conditions that are present only in B. thetaiotaomicron (Table 7). These loci include BT0523 to BT0524 coding for a sugar transferase with its cognate response regulator BT0532 encoding a anthranilate synthase component I family protein, BT0995 encoding a PAS domain-containing sensor histidine kinase, locus BT1042 to BT1045 encoding a SusD/RagB transporter system, BT1597 encoding a HAMP domain-containing histidine kinase (followed by a second component of the system and a gene coding for a cation efflux pump, BEX), and BT1895 to BT1896 encoding an outer membrane leucine-rich receptor system that is highly upregulated in colitis (Table 7).

BT2778, a sigma-70 family RNA polymerase sigma factor, the multigene locus BT3625 to BT3633 coding for a putative fimbrillin synthesis/transport locus, BT3969 encoding a heavy metal transporter, BT4227 encoding a MfaI fimbrillin protein, BT4236 encoding a two-component response regulator, and BT4771 encoding RNA polymerase ECF type sigma factor were present only in B. thetaiotaomicron (Table 7).

Some B. thetaiotaomicron loci had orthologs of selected genes present in other bacteria studied. Thus, the BT2063 to BT2065 locus was present mainly in B. thetaiotaomicron with the exception of an ortholog of the BT2065 coding for a TonB-dependent receptor also present in P. intermedia. BT2450 is the first gene in a locus coding for a hypothetical protein, pyrogenic exotoxin B (BT2451), and two hypothetical proteins (BT2452 to BT2453). *pdnA*, a gene similar to the BT2451, is present in P. gingivalis (PG1427) and codes for a periodontain light chain, as well as in P. intermedia (PIOMA14_I_1549) and encodes a C10 family peptidase (see Fig. S3 in the supplemental material). Also, while BT4233 was present only in B. thetaiotaomicron, BT4234 had an ortholog in P. intermedia.

Finally, highly upregulated in both studies (2) (high and low iron levels referring to the present study and the colitis study described in reference 2) was the locus BT2473 to BT2482 fimbrillin-family protein, two cytochrome c biogenesis proteins (CcsA), porin, thiol oxidoreductase, and peptidase M75 (helix-turn-helix transcriptional regulator, and three hypothetical proteins with 69.4- to 323.7-fold upregulation) (Table 5 and 7, Fig. 3G).

There were downregulated pathways, both in our study and in the colitis study. The main downregulated locus was BT0552 to BT0553 coding for a glutamate synthase (*glt*). Also downregulated in both studies was gene coding for asparagine synthase. In addition, a nitrogen regulator encoded by BT0545 was significantly downregulated. While the above genes were found only in B. thetaiotaomicron, the downregulated operon coding for succinate dehydrogenase/fumarate reductase is present in all three bacteria studied (Table 7).

## DISCUSSION

Our parallel study of three bacterial species belonging to the phylum of *Bacteroidetes* gives insight into the iron homeostasis in those organisms. During infection and associated with that inflammation, iron is scarce and thus the conditions in the host resemble those of the iron-limited conditions as reported in our study (21–24). Most *in vitro* studies are performed under iron-sufficient conditions and thus our study provides a more accurate depiction of the adaptation to the host environment. We further verified the latter by comparing our results with results obtained from studies done *in vivo* using a colitis model (2). Although, we reported previously that the P. gingivalis iron-dependent transcriptome, this work further extends that study as well as compares it to other bacteria belonging to *Bacteroidetes* with the ultimate goal of having a consensus on iron-dependent regulation in this group of bacteria. We have reported previously P. intermedia proteins whose expression is affected by iron; however, this is the first comprehensive report on iron-dependent regulation in P. intermedia, and the data reinforce our findings published previously (16) as well as data from other labs (25). The B. thetaiotaomicron iron-dependent regulation remained relatively unknown prior to our study. Here, we show a comprehensive analysis of the stimulon using RNA derived from bacteria grown under iron-replete and iron-chelated conditions. The number of regulated genes is larger than that found in P. gingivalis and P. intermedia that possibly is due to the much larger size of the genome of B. thetaiotaomicron (26) as well as the fact that B. thetaiotaomicron libraries were sequenced using the HiSeq Illumina sequencer while the other two library sets were sequenced using the MiSeq Illumina sequencer, thus resulting in a much larger number of reads for the HiSeq Illumina sequencer.

We do see changes in gene expression encoding proteins mediating several pathways, as follows: iron/heme acquisition, metabolism, and oxidative stress. Many of those pathways are shared among the three bacterial species. One of the most dramatically altered pathways is metabolism where the most drastically upregulated one was a gene coding for flavodoxin (FldA). The FldA structure was first determined using the Escherichia coli FldA (27); however, we note that the P. gingivalis FldA is more similar to the *Desulfovibrio* FldA structure (28). Flavodoxins are electron transfer proteins that are present in variety of bacteria as well as in algae and lower plants (29–31). They are low-molecular-weight, flavin mononucleotide (FMN)-containing proteins that function as electron transfer agents in a variety of microbial metabolic processes. Electron transfer proceeds through the exposed dimethylbenzene ring of the bound coenzyme FMN that is then reduced to FMNH_2_. FldAs were isolated over a half century ago, first from *Cyanobacteria* and later from *Clostridia* (32, 33). The expression of FldA was shown to be induced under iron-limiting conditions; however, their expression can also be regulated by other stresses. Their biological role is similar to that of ferredoxins as FldA transfers electrons from donors to the electron transport chain components, starting with the Rnf complex, located in the bacterial cell membrane, thus creating a sodium proton gradient driving ATP generation (34). While ferredoxins (Frds) use iron-sulfur clusters to transfer electrons, FldA binds the cofactor flavin mononucleotide (FMN) and thus is advantageous over ferredoxin as it spares iron for other essential enzymes relying on iron for their activity as well as is more resilient for oxidative/nitrosative stress damage. While we noted drastically upregulated FldA, we also noted a concomitant reduction in the expression of Frds under iron-limiting conditions. Such iron-dependent expression was also noted in other organisms (29, 35). As inflammatory conditions lead to reduced iron levels as well as elevated oxidative stress mediators released by inflammatory cells, adaptation through the elevation of FldA and replacement of Frd seems like an intuitive strategy benefiting the persistence of the bacteria under such conditions. Our data may also have translational significance as FldA is highly upregulated in B. thetaiotaomicron in the colitis model and its transcript is very abundant in P. gingivalis identified in patients with periodontal disease (19). Such data point to a novel metabolic mechanism employed by P. gingivalis that relies more on iron-independent enzymes during active disease status and thus contrasts with the thus-far favored metabolic model that is employed by the bacterium when grown in a laboratory under iron-rich conditions (36). It is noteworthy that FldA being an enzyme with characteristics unique to bacteria and not present in higher eukaryotes may also be a potential target for the future development of therapeutic strategies (31, 37).

The other highly upregulated pathway includes an iron/heme acquisition mechanism. Here, the well-defined system, the hemin uptake operon *hmu*, was observed to be upregulated in all three bacterial species tested. While in P. gingivalis and B. thetaiotaomicron one operon/genome was identified, the P. intermedia OMA14 displayed an *hmuY*-only carrying locus on the large chromosome (Ch I) and *hmu* operon on its small chromosome (Ch II) (Table 3). Interestingly, in P. intermedia 17 the *hmu* loci are also duplicated and present on separate chromosomes, but the orphan *hmuY* is located on the small chromosome (ChII) while the complete operon is present in the ChrI (Fig. S1). The latter observation is consistent with the recent report from the Olczak laboratory (25, 38). We observed a drastic upregulation of the complete *hmu* operon in all the three bacteria as well as the orphan P. intermedia 17 *hmuY* gene. Such findings are also in line with our previous report on the iron-dependent proteome in P. intermedia (16, 17). It is noteworthy that this operon is also upregulated in B. thetaiotaomicron under colitis conditions compared with that in health animals (2). In periodontitis, hepcidin levels leading to a reduction of systemic iron are elevated (21, 39). Accordingly, in periodontitis, microbial iron uptake mechanisms are significantly upregulated compared with healthy sites (40, 41). While iron acquisition through the transport of hemin using the *hmu* system would seem like an intuitive strategy under low extracellular iron conditions, we also observed an upregulation of two putative transporter systems that include TonB-dependent proteins and/or ABC-type transporters. The high upregulation under iron-deficient conditions of those systems in most of the bacterial species tested warrants further investigation in their role in mediating the growth and survival of the bacteria under low iron conditions. One such system, namely, BT2063 to BT2065, has been characterized recently in B. thetaiotaomicron and shown to play a crucial role in iron acquisition through the transport of xenosiderophores produced by Salmonella enterica serovar Typhimurium (2). This locus is the most drastically upregulated in B. thetaiotaomicron and has no similarity (orthologs) in the other two bacteria studied. It is also noteworthy that this is the most upregulated locus in B. thetaiotaomicron derived from an inflammatory condition (colitis model) compared with that in the non-inflamed host (2), thus further proving the biological significance of our findings.

The high upregulation of the loci with their composition, including ABC type transporters and/or TonB dependent proteins, also points to their role in possible iron acquisition from siderophores abundant in the oral or gut microbiomes. For the oral bacteria, such as P. gingivalis and P. intermedia, the acquisition of iron from lactoferrin abundant in the oral environment would seem like a probable strategy (3, 42). It would also be substantiated by the findings that many bacterial siderophores that have been purified have a higher affinity for iron Fe(III) than lactoferrin (43–46) under *in vitro* conditions. It is thus probable that the ability to “steal” siderophores produced by other bacteria is present in both P. gingivalis and P. intermedia as those are bacteria residing in the complex oral microbiome where siderophore providers are abundant (43).

We found proteins within a multigene locus with similar functions across the three bacteria studied despite them bearing low identity at the amino acid level. Examples of this finding may be the CdhR-carrying locus. Furthermore, the order of genes within loci may have differed. An example is the locus PG1180 to PG1181 coding for a transporter and an associated regulator that differ among the bacteria tested. The distribution of the genes within the locus differs as well, and thus, looking at pathways that are regulated in the context of a genome rather than searching for individual genes was a better prediction of the mechanisms that are deregulated during adaptation to low iron conditions.

Differences between the organisms were also detected and included mainly the regulation of genes mediating carbohydrate utilization in B. thetaiotaomicron. This result may be due to the different niches in which the bacteria live, as B. thetaiotaomicron relies on carbohydrate utilization (47). *Bacteroidetes* have elaborate enzyme combinations that they use for glycan breakdown (48). P. gingivalis and P. intermedia have smaller genomes with 2.3 Mb and 2.2 to 2.8 Mb (plus a minigenome ranging from 0.6 to 0.9 Mb), respectively, while the size of the genome of Bacteroides thetaiotaomicron is 6.26 Mb (VPI-4582 strain) and, thus, is over twice as large (26, 49–51). P. gingivalis lacks genes coding for glycan-degrading enzymes, and thus, the bacterium relies on peptides for energy generation (27, 49). It was noteworthy that the downregulation of TonB-dependent mechanisms was observed in P. intermedia and B. thetaiotaomicron under iron-limiting conditions. Such an observation opposes the predicted outcome of the overexpression of TonB-dependent mechanisms under iron-scarce conditions. However, it is in agreement that those mechanisms may transport other nutrients and, here, are predicted to be involved in glycan transport (47, 52).

While all bacteria examined were able to reduce nitrite, that ability was iron dependent in B. thetaiotaomicron. Such a finding is also in agreement with recent findings reported by Goff et al. (53). Furthermore, all bacteria tested have the nitrite reductase system NrfAH, and only the B. thetaiotaomicron operon is drastically downregulated under iron-limited conditions. We thus hypothesize that this operon plays a major role in the physiology of that bacterium. Although the reduction of expression and nitrite consumption was observed in the bacterium grown in the presence of an iron chelator, B. thetaiotaomicron is still able to consume large amounts of nitrite under iron-limiting conditions. Such a capability may assist not only B. thetaiotaomicron but also P. gingivalis and P. intermedia to detoxify toxic by-products of nitrate reduction to survive in the host. The nitrite levels in the oral cavity have been shown to exceed 1 mM, especially after a nitrate-rich meal (54–56). Furthermore, one of the host’s defense mechanisms deployed by immune cells is the production of nitric oxide that in turn can be oxidized to nitrite, and thus, effective nitrosative stress detoxification mechanisms would be expected in those bacteria.

We also detected a large number of small genes that were regulated by iron. The role of those genes, coding for DUF1661 domain-containing proteins, is yet to be established. It is noteworthy that those genes are specific to P. gingivalis, as no similarity to those genes in other bacteria was detected.

The striking similarity between genes upregulated under iron-limited conditions in our study and the genes upregulated in colitis as opposed to healthy subjects points to iron being the master regulator of *Bacteroides* adaptation to the host inflammatory condition. So far, knowledge of the molecular mechanisms regulating the expression of the effector proteins required for adaptation of *Bacteroidetes* to differing iron levels remains scarce. The identification of the above commonly regulated genes in all the three bacterial species is predicted to help identify iron-dependent regulatory systems in those bacteria. CdhR has been shown to regulate the expression of *hmuYR* (20, 57); however, the molecular mechanisms of the regulation remain unclear, as CdhR has yet to be shown to bind iron. Furthermore, given the clinical correlation between genes expressed under iron limited conditions and genes highly expressed during active inflammatory disease, the mechanisms identified in our study to be drastically upregulated gain biological significance, which warrants a further detailed investigation of their function and molecular mechanisms.

In conclusion, we have compared the iron-dependent stimulons of three bacterial species belonging to the *Bacteroidetes* phylum. In addition to the iron uptake mechanisms, the bacteria exhibited major adaptation of their metabolic mechanisms as well as oxidative stress response mechanisms. The observation that iron-limited conditions resembled those found in an inflamed host is consistent with data reported over several decades demonstrating iron sequestration as an innate immune response to bacterial infection (21–24). This result overall indicates that iron is the major player in the regulation of bacterial adaptation to diseased conditions. Ultimately, a more in-depth investigation of the molecular mechanisms of the iron-based effector proteins as well as the regulatory mechanisms that thus far remain elusive in the *Bacteroidetes* phylum is warranted.

## MATERIALS AND METHODS

### Bacterial strains and growth conditions.

For our study, we have used the following three bacterial species: Porphyromonas gingivalis (Strain W83) (58), Prevotella intermedia (strain OMA14), and Bacteroides thetaiotaomicron (strain BT5482 Δ*tdk*). Two of the species are oral bacteria, while B. thetaiotaomicron is a gut bacterium. All bacteria are clinically significant, and the strain P. intermedia OMA14 is a clinical isolate that has been sequenced recently (49). We also include a laboratory strain of P. gingivalis, namely, the ATCC 33277 strain, for comparison with the more virulent strain W83. The bacteria were maintained on blood agar plates (TSA II with 5% sheep blood; BBL, Cockeysville, MD) in an anaerobic chamber (10% H_2_, 10% CO_2_, and 80% N_2_) at 37°C. Broth cultures were prepared using enriched brain heart infusion (BHI) medium (37 g/L BHI, 2.5 g/L yeast extract, 5 μg/mL hemin, and 0.5 μg/mL menadione). Cultures were maintained under anaerobic conditions and grown at 37°C without agitation (static cultures). Iron-deplete conditions were generated by supplementing BHI with 150 μM dipyridyl (DP). Cells were harvested in mid-log phases (at an optical density at 600 nm [OD_660_] of approximately 0.5).

### RNA isolation and library preparation.

RNA isolation and library preparation were done as described previously (59, 60). Briefly, the RNeasy minikit (Qiagen) was used to isolate RNA from bacterial pellets following the manufacturer’s protocol. The RNA was treated with the DNA-free DNase kit (Ambion, Thermo Fisher) the following manufacturer’s protocol to remove any remaining DNA. The quality of RNA was verified using agarose gel electrophoresis to ensure that the RNA was intact and was then used to generate an RNAseq library using the Ovation complete prokaryotic RNASeq DR multiplex kit (Universal Prokaryotic RNAse multiplex kit [Tecan]) following the manufacturer’s instruction. The libraries were submitted to the Virginia Commonwealth University (VCU) Nucleic Acid Sequencing core facility where the quality of the libraries was verified using the bioanalyzer (Agilent Technologies).

### High-throughput sequencing and data analysis.

Libraries that were verified and had acceptable quality were sequenced by the (VCU) Nucleic Acid Sequencing core using the MiSeq Illumina genome analyzer (P. gingivalis and P. intermedia libraries) and with the Illumina HiSeq 4000 platform (B. thetaiotaomicron). The sequencing data were then analyzed with the CLC Genomic Workbench V12 (CLC Bio, Qiagen). Thus, the resulting transcriptome-derived sequences were aligned to the reference genomes of P. gingivalis W83 (for P. gingivalis W83 transcriptome), P. gingivalis ATCC 33277 (for P. gingivalis ATCC 33277 transcriptome), P. intermedia OMA14 (for the P. intermedia OMA14 transcriptome), and B. thetaiotaomicron VPI BT5482 (for the B. thetaiotaomicron VPI BT5482 Δ*tdk* transcriptome). The number of reads for each annotated gene was extracted and used for statistical analysis as described below.

### Bioinformatics analysis.

The BioCyc genome viewer was used to align genomic loci for selected genes (61). The alignment of similar loci was done across the three different bacterial species used in our study. The reference genomes used were P. gingivalis W83, P. intermedia 17 (the P. intermedia OMA14 genome is not available on BioCyc), and B. thetaiotaomicron BT5482.

### Nitrite reduction assay.

Bacterial strains were maintained on blood agar plates. Assays were done in BHI broth supplemented with 0.5 mg/mL cysteine, 5 μg/mL hemin, and 0.5 μg/mL menadione. Iron-deplete conditions were generated by supplementing the media with 150 μM dipyridyl (DP). Nitrite was added at 0.3 and 2 mM to *P. gingivalis/P. intermedia* and *B. thetaiotaomicron*, respectively. Actively growing, overnight cultures prepared in BHI broth were diluted 1:30 (for P. gingivalis W83 and P. intermedia OMA14) or 1:50 (for B. thetaiotaomicron) and grown anaerobically for 72 h at 37°C. Next, 1-mL aliquots were removed at different time points and used to determine bacterial growth and levels of nitrite. As a control, iron-replete (without DP) and iron-depleted media (supplemented with DP) with and without nitrite were maintained in parallel with the bacterial cultures.

Bacterial growth was monitored by measuring optical density at 660 nm, and nitrite levels were determined using the Griess reagent according to the manufacturer’s instructions (Abcam).

### Statistical analysis.

All experiments were repeated on at least four separate days (biological replicates). For the analysis of the changes at the levels of mRNA under iron-replete and iron-depleted conditions, the fold change and *P* values were derived from the four biological replicates. Bacterium-specific and gene-specific reads were used for the analysis. The enrichment for the iron-dependent regulation was determined by dividing the number of reads derived from the bacteria grown in iron-depleted conditions to that derived from the bacteria grown under iron-replete conditions for each position of the genome following normalization (59, 60, 62). The statistical analysis for the RNAseq data was carried out using the program and default settings available on the CLC Workbench 12 under the Toolbox|Workflows|RNA statistics workflow (Qiagen). Briefly, the empirical analysis of DGE (EdgeR) as outlined by Robinson et al. (63) was used for the analysis. The test is designed for samples where many features are examined simultaneously with limited biological replicates. Raw counts, which in turn were normalized and transformed, were used in the analysis. Two-group unpaired comparisons were carried out using reads per kilobase per million (RPKM) (RPKM is defined as the number of reads that are assigned to the transcript and divided by the transcript length and normalized by mapped reads) values (64). The *t* test was used to carry out the statistical analysis for the RNAseq data. Also, the *t* test was used to determine statistical significance for the nitrite assays done using data derived from at least three biological replicates of cultures prepared under iron-depleted and iron-replete conditions.

### Data availability.

The RNAseq data have been deposited to the NCBI Gene Expression Omnibus (GEO). The data are available under the accession numbers GSE169260 (for B. thetaiotaomicron BT5482 Δ*tdk*), GSE168982 (for P. intermedia OMA14), GSE168570 (for P. gingivalis W83), and GSE174493 (for P. gingivalis ATCC 33277).
