# Supplementary material for: Iron Deficiency Modulates Metabolic Landscape of Bacteroidetes Promoting Its Resilience during Inflammation
Source: Microbiol Spectr. 2023 Jun 14;11(4):e04733-22. doi: 10.1128/spectrum.04733-22 (PMC10434189; doi:10.1128/spectrum.04733-22)
Supplement: Supplemental file 1 — Supplemental material. Download spectrum.04733-22-s0001.pdf, PDF file, 0.6 MB [file spectrum.04733-22-s0001.pdf]

Supplementary Figures and Tables

- Iron-regulated loci in *Prevotella intermedia* OMA14

and other interesting loci in Bacteroidetes

- Genes upregulated and downregulated in *P. gingivalis* 33277

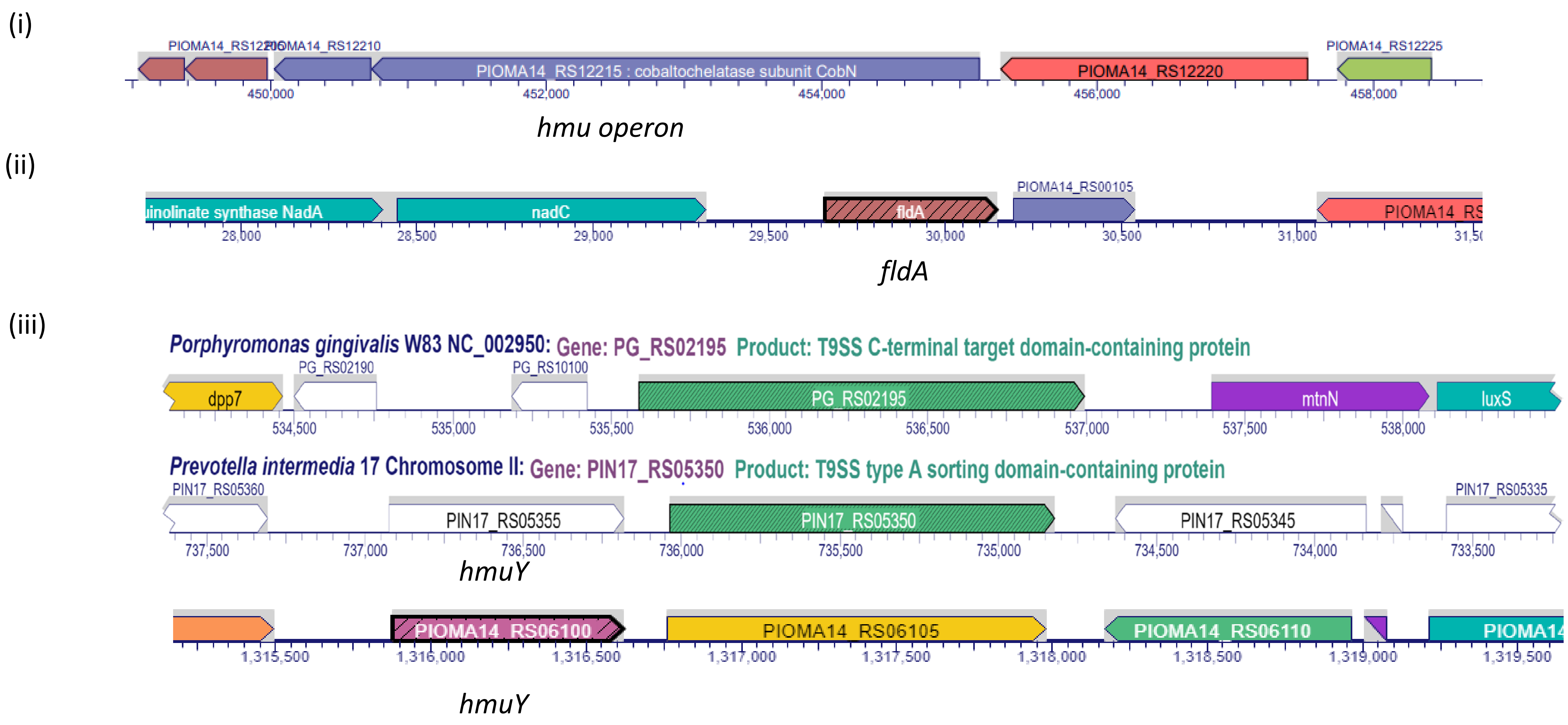

Supplemental Figure 1. Iron-regulated loci in *Prevotella intermedia* OMA14. A. Loci upregulated in low iron. *i. hmu* – full operon. *ii. fldA* – locus (PIOMA14_I_0020). iii. RS06100 *hmuY* (PIOMA14_I_1196)– one gene operon comparison with the same locus in *P. intermedia* 17 and *P. gingivalis* W83.

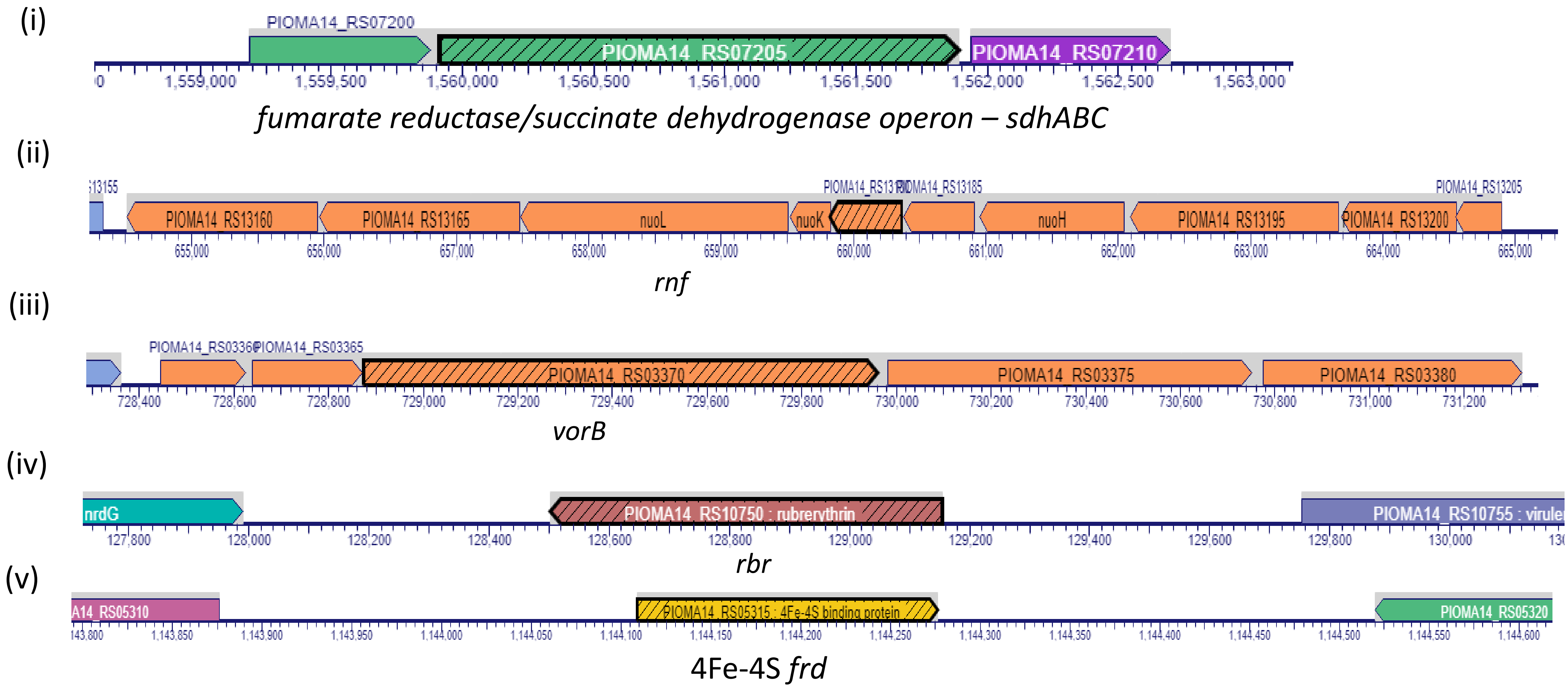

Supplemental Figure 1. Iron-regulated loci in *Prevotella intermedia* OMA14. B. Loci downregulated in low iron. i. *sdh* – locus (located on chromosome I), PIOMA14_I_1410-12, ii. RS13160-13200 *rnf* electron transport – PIOMA14_I_0607 – 0616, iii. PS03360-80 *vor* locus - PIOMA_I_0666 – 0670. iv. RS10750 *rbr* locus – PIOMA14_II_0105. v. RS05315 *frd* (4Fe-4S binding protein) – PIOMA14_I_1049.

(i)

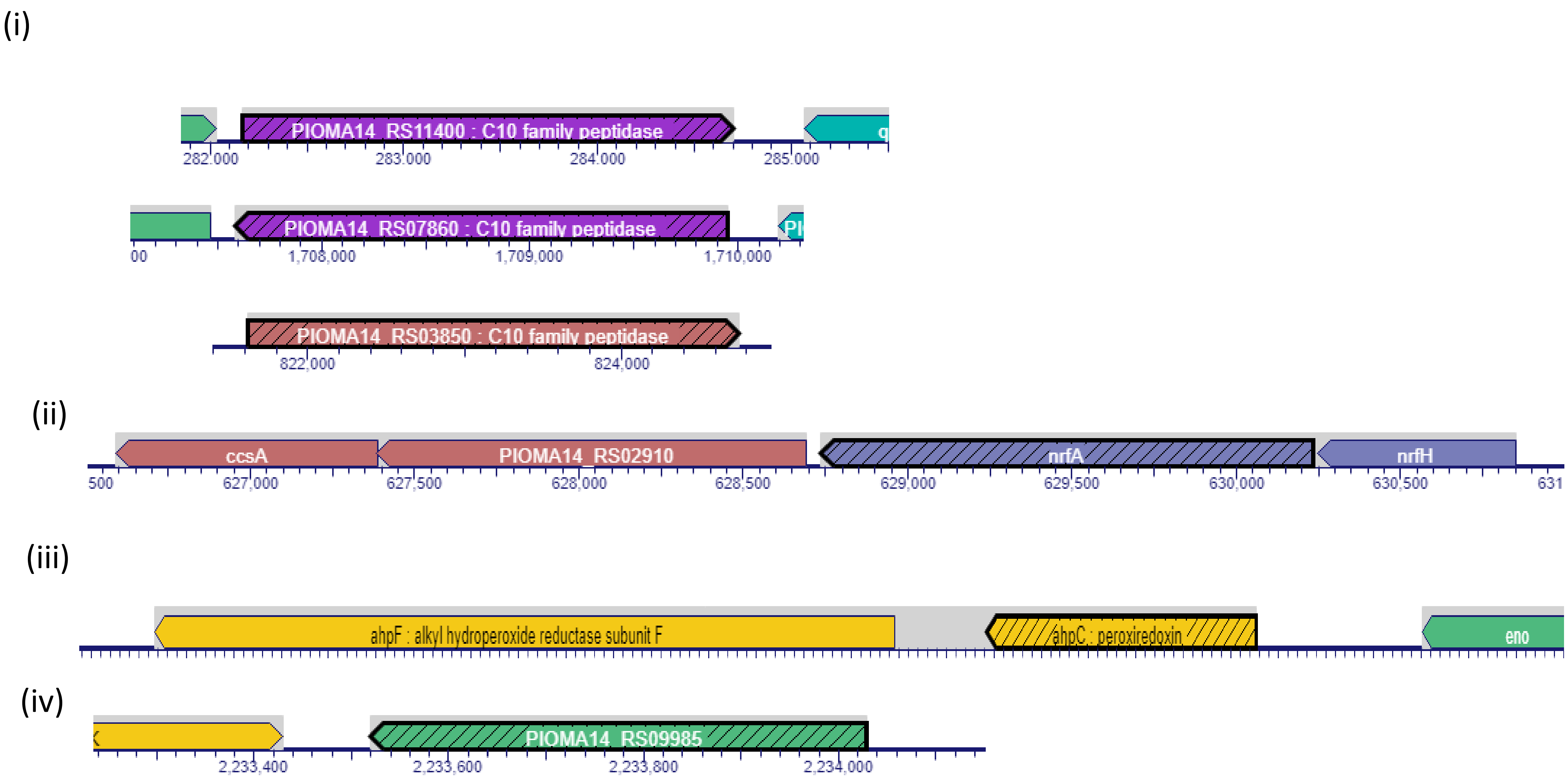

Supplemental Figure 2. Other loci of interest in *P. intermedia* OMA14. i. C10-family peptidase coding genes. ii. *nrfAH* locus. PIOMA14_I_0579 – 0582. RS00350 – 355 *ahpCF* locus – PIOMA14_I_0069 – 70. RS09985 *dps* locus PIOMA14_I_1962.

i.

Organism	Gene	Gene Product	Operon
B. thetaiotaomicron VPI-5482	BT_2451	pyrogenic exotoxin B	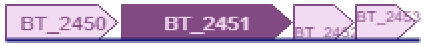
P. gingivalis W83	pdnA	periodontain light chain	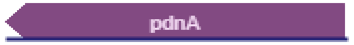
P. intermedia 17	PIN17_RS08020	C10 family peptidase	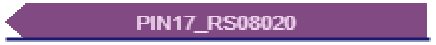

ii.

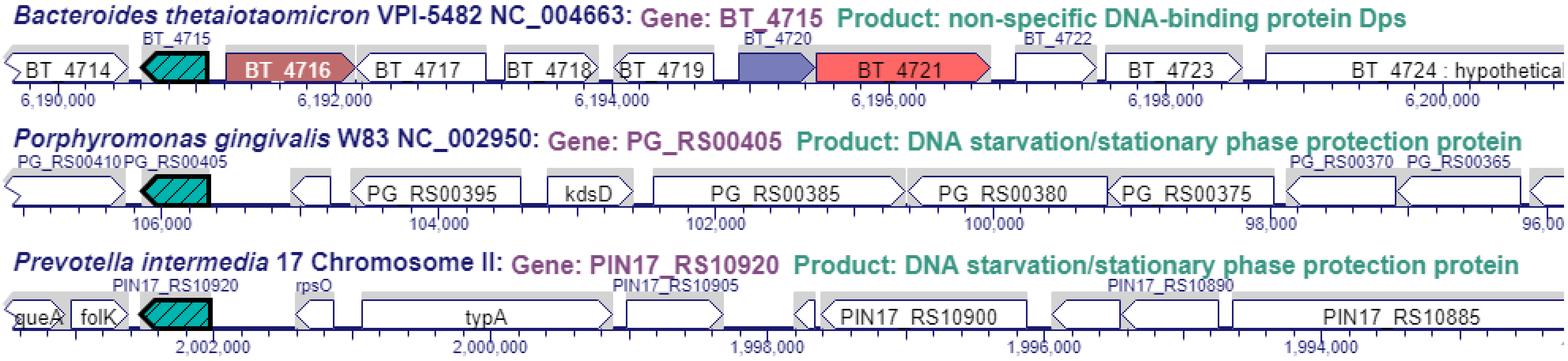

Supplemental Fig 3. Other interesting loci in Bacteroidetes. i. toxin/peptidase encoding gene. ii. *dps* coding for the DNA starvation/stationary phase protection protein.

A.

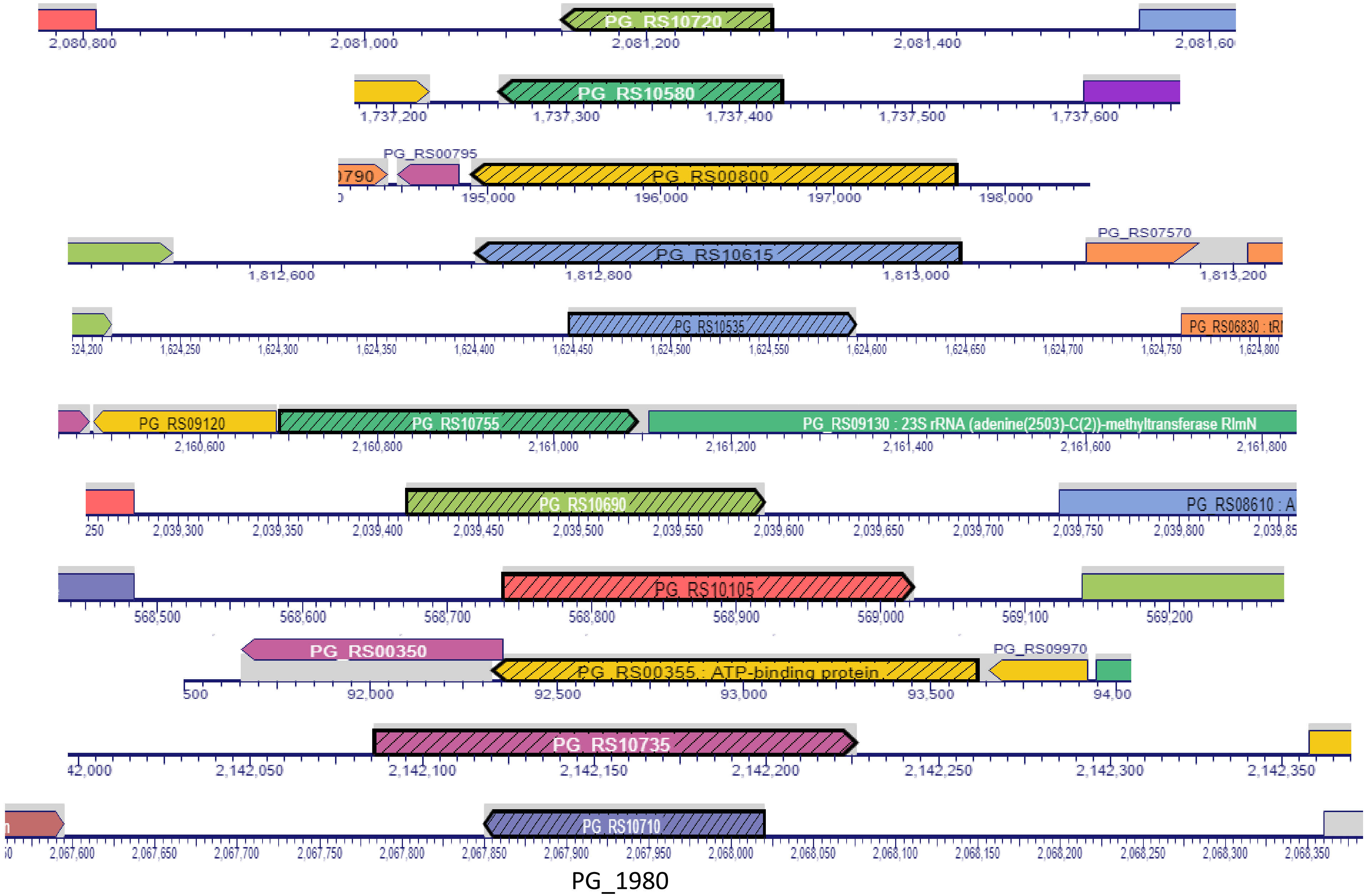

Supplemental Figure 4. Small proteins in Bacteroidetes. *P. gingivalis* W83 DUF1661 domain-containing proteins.
A. Genomic maps of the loci.

B.

Organism (Database) ▲ ▽	Accession ▵ ▽	Gene ▵ ▽	Product ▵ ▽	Found Based on ▵ ▽
Porphyromonas gingivalis W83	PG_RS10720	PG_RS10720	DUF1661 domain-containing protein	Same name/synonym
Porphyromonas gingivalis W83	PG_RS10580	PG_RS10580	DUF1661 domain-containing protein	Same name/synonym
Porphyromonas gingivalis W83	PG_RS00800	PG_RS00800	DUF1661 domain-containing protein	Same name/synonym
Porphyromonas gingivalis W83	PG_RS10615	PG_RS10615	DUF1661 domain-containing protein	Same name/synonym
Porphyromonas gingivalis W83	PG_RS10535	PG_RS10535	DUF1661 domain-containing protein	Same name/synonym
Porphyromonas gingivalis W83	PG_RS10755	PG_RS10755	DUF1661 domain-containing protein	Same name/synonym
Porphyromonas gingivalis W83	PG_RS10690	PG_RS10690	DUF1661 domain-containing protein	Same name/synonym
Porphyromonas gingivalis W83	PG_RS10105	PG_RS10105	DUF1661 domain-containing protein	Same name/synonym
Porphyromonas gingivalis W83	PG_RS09970	PG_RS09970	DUF1661 domain-containing protein	Same name/synonym
Porphyromonas gingivalis W83	PG_RS10735	PG_RS10735	DUF1661 domain-containing protein	Same name/synonym
Porphyromonas gingivalis W83	PG_RS10710	PG_RS10710	DUF1661 domain-containing protein	Same name/synonym and Ortholog

Supplemental Figure 4. Small proteins in Bacteroidetes. *P. gingivalis* W83 DUF1661 domain-containing proteins. B. List of DUF1661 domain-containing proteins.

C.

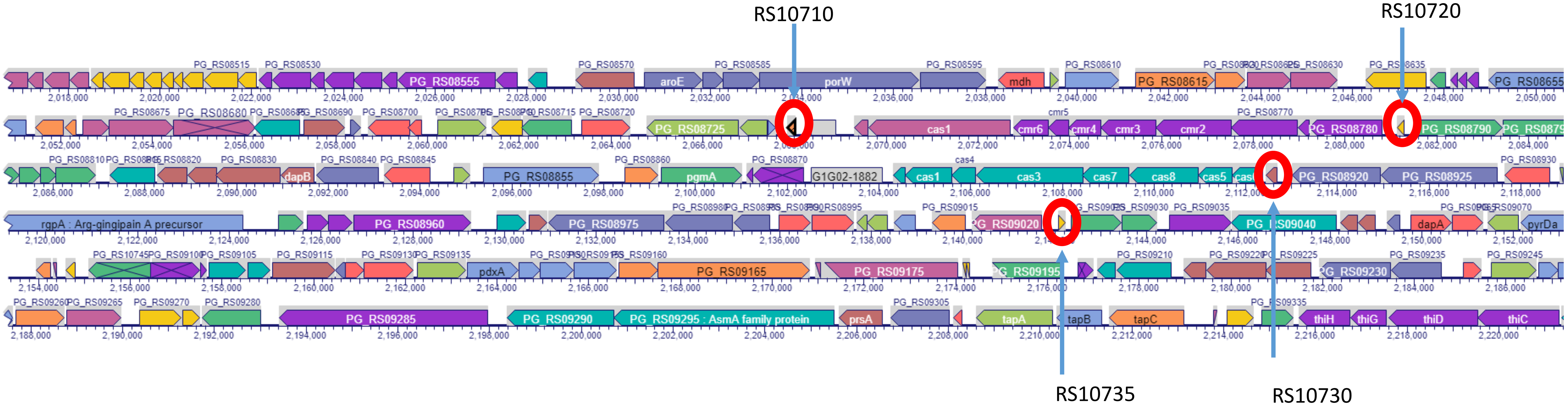

Supplemental Figure 4. Small proteins in Bacteroidetes. *P. gingivalis* W83 DUF1661 domain-containing proteins. C. Location of selected DUF1661 domain-containing proteins on a genome of *P. gingivalis* W83.

D.

PG1980	Query_87146	1	MVREVKNSRATAKKFSRRFSRKHAPQSEHFRSVNFGEadLSEKESS[10]	56
PG1980	AAQ66953.1	1	MVREVKNSRATAKKFSRRFSRKHAPQSEHFRSVNFGEadLSEKESS[10]	56
	WP_099841637.1	1	MAWEAKNSHATTKKFSRHFFREYAPQSEHFRFVFF-----------	35
	WP_143733541.1	1	MVRLFFFSRAATKKFSRRFSGKHAPQSERFRPENFILplFDNYPFP[7]	53
	WP_080502166.1	1	[19]LVREAKNSRATTKKFSRLFFRILRPQSEHFPFV------FSRKQIS	59
	WP_230456049.1	1	[8]LVREVKNLRAKTKKFSCVFSRKHEPQSARFRRDFFI----------	44
	AAQ65412.1	1	MVREAKNLRARTKKISLHICRKHAPQSEDFRCVFLRQqvIDRDIEA[892]	938
	AAQ67025.1	1	[12]LVREVKNLRATAKKISRHFFRKYEPQPGVFWFV-FHLsrSHPFPFL[77]	134
	WP_130267169.1	1	MVREAKNSRAKAKKFSRRFSGI------------------------	22
	WP_143734319.1	1	MAREVKIFGTKTKNFSRVFSPKHAPQSDRFWLVIFRQ---------	37
	WP_230456104.1	1	----MKNSRATTKKFRRYFWEKHRPQF-------------------	23
	WP_230456000.1	1	----MKKTRAGTKKLRFHFFRKHVPQSERFRFVFLCWqvMSMCLQG	42
	WP_144043238.1	1	[12]LVREVKNSRATRKKFS------------------------------	28

Supplemental Figure 4. Small proteins in Bacteroidetes. *P. gingivalis* W83 DUF1661 domain-containing proteins.
D. Multiple alignment of the protein sequences.

Supplemental Table 1. Genes upregulated at least 2 fold in *Porphyromonas gingivalis* ATCC33277 under iron deplete conditions

[1]Fold change	[2]P-value	Locus_tag (NC_010729 (CDS))	Old_locus_tag (NC_010729 (CDS))	Product (NC_010729 (CDS))
2.000584	0.007688	PGN_RS03120	PGN_0656	hypothetical protein
2.021253	0.002067	PGN_RS09245	PGN_1953	TonB-dependent receptor
2.035311	0.011206	PGN_RS02340, PGN_RS02345	PGN_0490, PGN_0491	MATE family efflux transporter, low molecular weight phosphotyrosine protein phosphatase
2.061333	0.015305	PGN_RS01770	PGN_0373	redoxin domain-containing protein
2.093161	0.068863	PGN_RS10600		ABC transporter permease
2.096857	0.01872	PGN_RS08255	PGN_1738	AMP-binding protein
2.111029	0.00271	PGN_RS05390	PGN_1125	NfeD family protein
2.171439	0.006434	PGN_RS04475	PGN_0936	D-2-hydroxyacid dehydrogenase
2.203448	0.000793	PGN_RS04990	PGN_1041	cytochrome ubiquinol oxidase subunit I
2.216498	0.00763	PGN_RS08395	PGN_1767	T9SS type A sorting domain-containing protein
2.275778	0.014335	PGN_RS03125	PGN_0657	T9SS type A sorting domain-containing protein
2.366644	0.000339	PGN_RS06250	PGN_1308	DtxR family transcriptional regulator
2.373587	0.001769	PGN_RS08490	PGN_1789	dCMP deaminase family protein
2.385498	0.001336	PGN_RS09640	PGN_2037	DNA starvation/stationary phase protection protein
2.390405	0.003543	PGN_RS04945	PGN_1032	hypothetical protein
2.438962	0.280262	PGN_RS11100		DUF1661 domain-containing protein
2.489813	0.001588	PGN_RS11610		hypothetical protein
2.514346	0.000277	PGN_RS06550	PGN_1374	RluA family pseudouridine synthase
2.541556	0.000552	PGN_RS03435	PGN_0720	ABC transporter permease
2.568611	0.000181	PGN_RS02340, PGN_RS02345	PGN_0490, PGN_0491	MATE family efflux transporter, low molecular weight phosphotyrosine protein phosphatase
2.630542	0.008221	PGN_RS08265	PGN_1740	RNA polymerase sigma factor
2.748884	0.002757	PGN_RS01845	PGN_0388	thiol peroxidase
2.934543	2.64E-05	PGN_RS07670	PGN_1611	leucine-rich repeat domain-containing protein
3.162471	0.000495	PGN_RS04565	PGN_0955	IS982-like element IS195 family transposase
3.712825	3.7E-05	PGN_RS02350	PGN_0492	copper-translocating P-type ATPase
3.853486	3E-06	PGN_RS04075	PGN_0852	T9SS type A sorting domain-containing protein
3.985249	7.18E-05	PGN_RS03140	PGN_0660	peroxiredoxin
4.26831	0.000501	PGN_RS03145	PGN_0661	alkyl hydroperoxide reductase subunit F
4.402052	0.00011	PGN_RS02355	PGN_0493	heavy-metal-associated domain-containing protein
4.527286	3.31E-08	PGN_RS03445	PGN_0722	PAS domain-containing protein
4.817032	6.31E-06	PGN_RS09240	PGN_1951	hypothetical protein
7.326125	1.99E-09	PGN_RS06540, PGN_RS06545	PGN_1372, PGN_1373	hemerythrin domain-containing protein, response regulator transcription factor
7.955087	0.198373	PGN_RS11195		ISAs1 family transposase

8.150926	5.67E-12	PGN_RS11630		hypothetical protein
8.911972	5.3E-13	PGN_RS02625	PGN_0553	DUF2149 domain-containing protein
9.034596	4.24E-11	PGN_RS08525	PGN_1797	isoprenylcysteine carboxyl methyltransferase family protein
9.121851	5.22E-15	PGN_RS06540, PGN_RS06545	PGN_1372, PGN_1373	hemerythrin domain-containing protein, response regulator transcription factor
9.704682	0	PGN_RS02630	PGN_0554	MotA/TolQ/ExbB proton channel family protein
12.55265	0	PGN_RS02635, PGN_RS02640	PGN_0555, PGN_0556	hypothetical protein, cobaltochelatase subunit CobN
12.91995	0	PGN_RS02635, PGN_RS02640	PGN_0555, PGN_0556	hypothetical protein, cobaltochelatase subunit CobN
13.40529	0	PGN_RS02645	PGN_0557	TonB-dependent receptor
14.14122	0	PGN_RS08530	PGN_1798	class I SAM-dependent methyltransferase
18.16092	0	PGN_RS06370, PGN_RS06375	PGN_1334, PGN_1335	hypothetical protein, TonB-dependent receptor
19.33852	1.01E-12	PGN_RS04540	PGN_0951	hypothetical protein
26.19061	0	PGN_RS06370, PGN_RS06375	PGN_1334, PGN_1335	hypothetical protein, TonB-dependent receptor
29.84871	0	PGN_RS09080	PGN_1915	hypothetical protein
29.85613	0	PGN_RS02650	PGN_0558	HmuY family protein
38.18532	0	PGN_RS06380	PGN_1336	DUF4876 domain-containing protein
39.33577	0	PGN_RS04530	PGN_0949	ABC transporter ATP-binding protein
39.42041	0	PGN_RS07040	PGN_1476	T9SS type A sorting domain-containing protein
40.5205	0	PGN_RS09090	PGN_1917	ABC transporter ATP-binding protein
45.76565	0	PGN_RS08495	PGN_1790	DUF2023 family protein
54.26255	0.01426	PGN_RS06125	PGN_1281	conjugative transposon protein TraM
54.31768	0	PGN_RS04525	PGN_0948	hypothetical protein
55.38062	0	PGN_RS09110	PGN_1921	TetR/AcrR family transcriptional regulator
57.83104	0	PGN_RS09095	PGN_1918	hypothetical protein
63.10688	0	PGN_RS04535	PGN_0950	ABC transporter ATP-binding protein
63.29707	0	PGN_RS04520	PGN_0947	outer membrane lipoprotein-sorting protein
64.60049	0	PGN_RS04515	PGN_0946	MMPL family transporter
65.19743	0	PGN_RS09100	PGN_1919	outer membrane lipoprotein-sorting protein
65.58828	0	PGN_RS09085	PGN_1916	ABC transporter ATP-binding protein
68.18361	0	PGN_RS09105	PGN_1920	MMPL family transporter
92.89672	0	PGN_RS04510	PGN_0945	TetR/AcrR family transcriptional regulator
111.564	0	PGN_RS08500	PGN_1791	flavodoxin

[1]Ratio of gene expression in bacteria grown in iron deleted compared to iron rich conditions

[2]P value from an experiment performed in four independent biological replicates

Supplemental Table 2. Genes downregulated at least 2 fold in *Porphyromonas gingivalis* ATCC33277 under iron deplete conditions

[1]Fold change	[2]P-value	Locus_tag (NC_010729 (CDS))	Old_locus_tag [NC_010729 (CDS)]	Product (NC_010729 (CDS))
-18.4295	0	PGN_RS01450	PGN_0302	rubrerythrin family protein
-8.88831	0.026627	PGN_RS11215, PGN_RS11220		DUF1661 domain-containing protein
-5.89335	0.272045	PGN_RS10990		DUF1661 domain-containing protein
-4.80065	0.005033	PGN_RS09595	PGN_2027	DUF1661 domain-containing protein
-4.5836	0.020732	PGN_RS11045		DUF1661 domain-containing protein
-4.43088	0.364412	PGN_RS11525, PGN_RS10845		DUF1661 domain-containing protein
-4.39787	0.365009	PGN_RS06305	PGN_1319	IS5 family transposase
-4.39772	0.367454	PGN_RS00470, PGN_RS00475	PGN_0100, PGN_0101	diaminopimelate decarboxylase, 1,4-dihydroxy-2-naphthoate octaprenyltransferase
-4.18373	4.16E-08	PGN_RS08325	PGN_1752	4Fe-4S binding protein
-3.70525	0.07624	PGN_RS11580	PGN_0603	hypothetical protein
-3.68901	0.01228	PGN_RS11485		hypothetical protein
-3.6443	0.018009	PGN_RS10955		DUF1661 domain-containing protein
-3.52579	0.026314	PGN_RS04505	PGN_0944	IS982-like element IS195 family transposase
-3.3219	1.8E-06	PGN_RS08330	PGN_1753	3-methyl-2-oxobutanoate dehydrogenase subunit VorB
-3.27322	0.138822	PGN_RS10905		DUF1661 domain-containing protein
-3.18553	2.25E-05	PGN_RS08335	PGN_1754	hypothetical protein
-3.06841	1.03E-05	PGN_RS08340	PGN_1755	2-oxoglutarate oxidoreductase
-3.02946	1.43E-05	PGN_RS08345	PGN_1756	2-oxoacid:acceptor oxidoreductase family protein
-2.96769	0.004613	PGN_RS02735		site-specific integrase
-2.92239	0.026505	PGN_RS11075		DUF1661 domain-containing protein
-2.7624	0.044271	PGN_RS11260		DUF1661 domain-containing protein
-2.72016	0.025089	PGN_RS00505	PGN_0108	IS5/IS1182 family transposase
-2.70385	0.00153	PGN_RS04555	PGN_0953	IS982-like element IS195 family transposase
-2.66266	0.361949	PGN_RS11160		IS982 family transposase
-2.5566	0.000241	PGN_RS07840	PGN_1647	50S ribosomal protein L27
-2.5331	0.029003	PGN_RS11420		DUF1661 domain-containing protein
-2.52019	0.000687	PGN_RS10100	PGN_0166	transposase
-2.43839	0.058694	PGN_RS10240		DUF1661 domain-containing protein
-2.43199	0.008965	PGN_RS02740	PGN_0577	IS5 family transposase
-2.41758	0.02568	PGN_RS11550	PGN_0127	hypothetical protein
-2.41276	0.149781	PGN_RS11585		ISAs1 family transposase
-2.2942	0.008033	PGN_RS09355	PGN_1978	SRPBCC domain-containing protein
-2.28235	0.007726	PGN_RS07865, PGN_RS07870	PGN_1652, PGN_1653	nitroreductase family protein, FAD:protein FMN transferase
-2.24278	0.033729	PGN_RS10995		DUF1661 domain-containing protein

-2.19947	0.003578	PGN_RS07900	PGN_1659	Fe-S cluster domain-containing protein
-2.18742	0.228972	PGN_RS11615		DUF1661 domain-containing protein
-2.16609	0.052554	PGN_RS10420	PGN_0940	transposase family protein
-2.16443	0.003892	PGN_RS07880, PGN_RS07885, PGN_RS07890	PGN_1655, PGN_1656, PGN_1657	electron transport complex subunit E, RnfABCDGE type electron transport complex subunit G, RnfABCDGE type electron transport complex subunit D
-2.15577	0.014757	PGN_RS01675	PGN_0350	translation initiation factor
-2.14288	0.004314	PGN_RS07885, PGN_RS07890	PGN_1656, PGN_1657	RnfABCDGE type electron transport complex subunit G, RnfABCDGE type electron transport complex subunit D
-2.08918	0.003993	PGN_RS07880, PGN_RS07885	PGN_1655, PGN_1656	electron transport complex subunit E, RnfABCDGE type electron transport complex subunit G
-2.05641	0.006102	PGN_RS07895	PGN_1658	electron transport complex subunit RsxC
-2.03438	0.034948	PGN_RS10455	PGN_1007	DUF1661 domain-containing protein
-2.00761	0.030833	PGN_RS08765	PGN_1849	50S ribosomal protein L15

[1]Ratio of gene expression in bacteria grown in iron deleted compared to iron rich conditions

[2]P value from an experiment performed in four independent biological replicates
